# Ptbp1 Knockdown in Glial Cells Promotes Motor and Sensory Function Recovery After Peripheral Nerve Injury

**DOI:** 10.1111/cns.70531

**Published:** 2025-07-23

**Authors:** Honghao Song, Lei Peng, Dashuang Chen, Xiaoyi Fan, Tong Hua, Ruifeng Ding, Mengqiu Deng, Qianbo Chen, Mei Yang, Hongbin Yuan

**Affiliations:** ^1^ Department of Anesthesiology Changzheng Hospital, Naval Medical University Shanghai People's Republic of China; ^2^ Department of Anesthesiology and Intensive Care Third Affiliated Hospital of Naval Medical University Shanghai People's Republic of China

**Keywords:** astrocyte, nerve regeneration, peripheral nerve injury, polypyrimidine tract‐binding protein 1, satellite glial cell

## Abstract

**Background:** Peripheral nerve injury (PNI) frequently causes persistent sensory and motor deficits with limited therapeutic options. While Ptbp1‐mediated astrocyte reprogramming shows promise in central nervous system repair, its role in PNI—particularly regarding spinal cord astrocytes and dorsal root ganglia (DRG) satellite glial cells (SGCs)—remains unexplored.**Aims:** This study aimed to determine whether Ptbp1 knockdown in glial cells enhances functional recovery after sciatic nerve injury (SNI) by dual mechanisms: (1) converting spinal cord astrocytes to motor neurons and polarizing them toward neuroprotective A2 phenotype, and (2) activating regenerative signaling pathways in DRG SGCs.**Materials & Methods:** C57BL/6J mice underwent SNI followed by intrathecal injection of AAV‐GFAP‐CasRx‐Ptbp1 (targeting Ptbp1 in astrocytes/SGCs) or control virus. Primary astrocytes and SGCs were transfected with Ptbp1 siRNA in vitro. Assessments included functional recovery (Basso Mouse Scale, Louisville Swim Score, Hargreaves test, von Frey assay), axonal regeneration (HE/β3‐tubulin/SCG‐10 staining), transcriptome/ATAC sequencing, and molecular analyses (immunofluorescence for DCX/Islet1/ntng2‐NGL‐2; Western blot for Ptbp1/GDNF/C3).**Results:** Ptbp1 was upregulated in spinal cord astrocytes and DRG SGCs post‐SNI. Its knockdown accelerated motor/sensory functional recovery and axonal regeneration. Mechanistically, in the spinal cord, Ptbp1 depletion induced astrocyte‐to‐motor neuron conversion (upregulation of DCX/Islet1/Map2) and polarized astrocytes toward A2 phenotype (upregulation of S100a10/GDNF; downregulation of C3). In DRG, it activated the ntng2/NGL‐2 pathway in SGCs, enhancing sensory axon regeneration (upregulation of ATF3/GAP43). Ntng2 blockade abolished sensory regeneration, confirming pathway dependence.**Discussion:** Ptbp1 knockdown promotes PNI repair through spatially distinct mechanisms: spinal cord astrocyte reprogramming/A2 polarization synergizes with DRG SGC‐mediated ntng2/NGL‐2 activation. While astrocyte‐to‐neuron conversion was limited, dominant A2 polarization provided neuroprotection. The absence of SGC transdifferentiation highlights cell‐type‐specific responses. Limitations include low conversion efficiency and interspecies regenerative differences.**Conclusion:** Targeting Ptbp1 in glial cells accelerates PNI recovery by dual regenerative mechanisms: motor function restoration via astrocyte‐derived neuron replenishment and A2 polarization, coupled with sensory repair through ntng2/NGL‐2 pathway activation. This establishes Ptbp1 as a promising therapeutic target for nerve injuries.

## Graphical abstract

Knockdown of Ptbp1 in spinal cord astrocytes promotes motor function recovery after sciatic nerve injury by inducing their transdifferentiation into motor neurons and polarization toward the neuroprotective A2 phenotype. Concurrently, Ptbp1 depletion in dorsal root ganglion satellite glial cells enhances sensory axon regeneration through the activation of the ntng2/NGL‐2 signaling pathway.

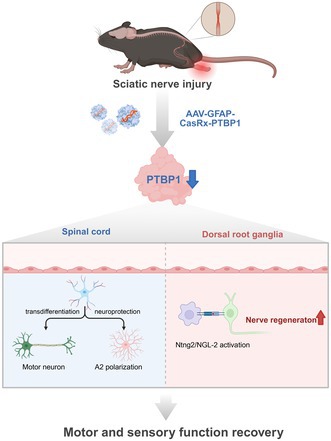

**Abbreviations**
AAV: adeno‐associated virus
DCX: doublecortin
DRG: dorsal root ganglia
EDTA: ethylenediaminetetraacetic acid
GDNF: glial cell‐derived neurotrophic factor
GFAP: glial fibrillary acidic protein
IL‐1β: interleukin‐1β
NGL‐2: netrin‐g2 ligand
ntng2: netrin‐g2
RIPA: radioimmunoprecipitation assay
RNA‐seq: RNA sequencing
SNI: sciatic nerve injury
WB: Western blot

## 1 Introduction

Following an injury to the PNS, functional recovery often occurs due to the inherent ability of peripheral neurons to undergo self‐repair and reactivate intrinsic growth programs in the aftermath of the injury [1]. Nevertheless, severe trauma to peripheral nerves may result in enduring neurological deficits, encompassing reinnervation failure and the onset of persistent pain [2, 3]. Damage to the PNS affects nerves located outside the central nervous system. This includes the full complement of cranial nerve pairs, the complete set of spinal nerve pairs, and the entirety of the autonomic nerves [4]. Among these injuries, sciatic nerve injury (SNI) serves as a paradigmatic illustration of PNS injury, characterized by the disruption of nerve fiber continuity [5]. The sciatic nerve consists of motor fibers originating from motor neurons in the anterior horn of the spinal cord and sensory fibers from sensory neurons in the DRG, making it an ideal system for studying the different roles and interconnections of these structures in nerve repair and function recovery [6, 7, 8, 9]. Recently, the research on the mechanism of cellular and molecular response after injury [1], the development process of the nervous system [10], regenerative organisms [11], and molecular screening technology [12] has revealed various mechanisms influencing regenerative potential. Further efforts need to be focused on the mechanism and treatment of nerve repair.

Recently, in the field of regenerative medicine, supplementation of newborn neurons has been suggested to be an ideal method for nerve injury repair [13]. In vivo reprogramming technology has emerged as a promising means of promoting neuronal regeneration [14, 15]; thus, promoting neuronal regeneration for peripheral nerve injury repair appears to be a viable mechanism. Several molecules can effectively convert astrocytes into neurons, with Ptbp1 being one of the most extensively studied of these molecules [16]. In recent in vitro and in vivo research, a subtraction strategy to convert astrocytes into neurons by depleting Ptbp1 was developed. Due to its simplicity, this approach has attracted significant attention, prompting numerous research groups to validate its findings and explore potential expansions. However, they encountered difficulties in tracing the lineage of newly generated neurons derived from astrocytes, suggesting that neuronal egress could be another explanation for the observed transdifferentiation of astrocytes to neurons [16, 17, 18, 19]. This challenge remains, but the fact that Ptbp1 depletion appears to reverse deficits in neurodegenerative disease by turning a specific subpopulation of glial cells into neurons, among other mechanisms, is noteworthy. While current studies have focused on the brain, whether Ptbp1 has a role in peripheral nerve injury needs to be further investigated.

In addition to the possibility of astrocyte transdifferentiation, we have concurrently focused on the possible effects of astrocyte status on nerve regeneration. Astrocytes are the predominant cell type in the spinal cord, essential for preserving homeostasis and regulating neuronal circuit activity [20]. Studies on the peripheral nervous system suggest that remote nerve damage elicits inflammatory reactions both locally and in remote neurons, even before immune cell infiltration occurs at the injury site [21]. Emerging evidence suggests that astrocytes are crucial mediators of neuroinflammation in nerve injuries and diseases [22]. They can become highly reactive, transforming into “reactive astrocytes” [23]. These cells may adopt either a cytotoxic A1 phenotype or a protective A2 phenotype [24]. Polarizing astrocytes toward the A2 type or facilitating the transition of the A1 phenotype to the alternative A2 phenotype are options for facilitating nerve repair.

Similarly, the DRG serves as a central hub for sensory nerve regeneration, facilitating axonal regrowth and signaling [25]. Within the DRG, satellite glial cells (SGCs) play a key role in modulating neuronal repair by altering their phenotype and interacting with neurons. Recent research indicates that the elevated expression of genes linked to regeneration, like ATF3 and GAP43, intrinsic to the neuronal injury response, partially depends on signaling from SGCs surrounding the neuronal soma. This suggests that neurons and their surrounding glial outer membrane may function as a coordinated unit to facilitate nerve repair [26]. However, the role of Ptbp1 knockdown in SGCs remains to be elucidated and warrants further exploration.

In the present study, we hypothesized that knocking down Ptbp1 would enhance sciatic nerve regeneration by modulating distinct but interconnected mechanisms in the spinal cord and DRG. Our findings reveal that Ptbp1 knockdown accelerates motor neuron axon regeneration by promoting astrocyte‐to‐neuron conversion and A2 polarization in the spinal cord while enhancing sensory axon repair by activating the ntng2/NGL‐2 signaling pathway in satellite glial cells. These results offer new insights into peripheral nerve regeneration mechanisms and highlight potential therapeutic targets.

## 2 Results

### 2.1 Increased Ptbp1 Expression in the Spinal Cord and DRG After SNI

**FIGURE 1:**
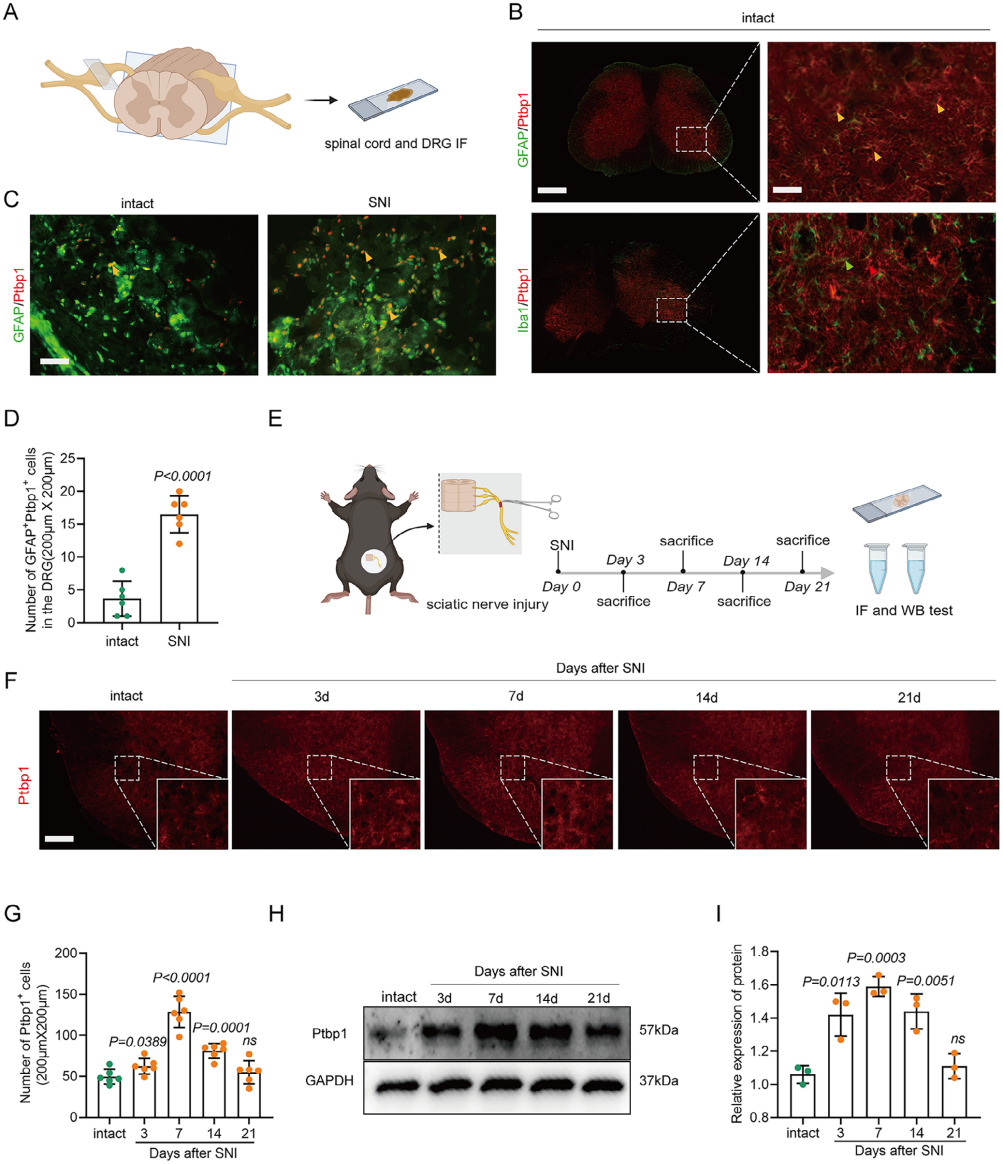
Ptbp1 expression is elevated in astrocytes and satellite glial cells in the SNI model. (A) Schematic of the experiment. (B) Immunofluorescence staining of Ptbp1 with GFAP and Iba‐1 in the spinal cord. Yellow arrowheads indicate the colocalization of Ptbp1 and GFAP. Scale bar, 200 μm. (C) Immunofluorescence staining of Ptbp1 with GFAP in the DRG. Scale bar, 100 μm. (D) Number of GFAP^+^Ptbp1^+^ cells in the DRG (*n* = 6 biological replicates per group; unpaired t test; mean ± SEM). (E) Schematic of the follow‐up experiment. (F) Immunofluorescence of Ptbp1 expression in the spinal dorsal horn at different time points after SNI. Scale bar, 100 μm. (G) Number of Ptbp1^+^ cells in the spinal cord (*n* = 6 biological replicates per group; unpaired t test; mean ± SEM). (H, I) Western blotting (H) and quantification (I) were used to determine the protein level of Ptbp1 in the spinal cord at different time points after SNI (*n* = 3 biological replicates per group; unpaired *t* test; mean ± SEM).

In the spinal cord, the primary glial cells identified are astrocytes and microglia, as evidenced by existing literature [27]. To elucidate the function of Ptbp1 in a SNI model, our initial approach involved pinpointing the cellular localization of Ptbp1 within the spinal cord and DRG via immunofluorescence staining (Figure 1A). The immunofluorescence analysis demonstrated that Ptbp1 in the spinal cord co‐localized with GFAP and not with Iba1, indicating that Ptbp1 was expressed mainly in astrocytes (Figure 1B), as confirmed statistically (Figure S1). Additionally, it was found that Ptbp1 colocalized with GFAP, which indicated that Ptbp1 was primarily expressed in satellite glial cells within the DRG. Moreover, the substantial upregulation of Ptbp1 expression was observed in the DRG post‐SNI (Figure 1C) with statistical significance (Figure 1D).

To further substantiate the temporal dynamics of Ptbp1 expression, we employed immunofluorescence staining and Western blotting to evaluate Ptbp1 expression at various time points post‐SNI (3rd, 7th, 14th, and 21st day) (Figure 1E). Immunofluorescence staining revealed that Ptbp1 expression substantially increased on the 3rd, 7th, and 14th days post‐modeling (Figure 1F) with statistical significance (Figure 1G). This is further confirmed by the pronounced increase in Ptbp1 protein expression on the 7th and 14th days post‐modeling (Figure 1H), as confirmed statistically (Figure 1I). Collectively, these findings imply that Ptbp1 expression is predominantly localized in astrocytes and satellite glial cells, and is substantially upregulated during the pathological progression of SNI.

### 2.2 Efficient Knockdown Expression of Ptbp1 in Spinal Cord Astrocytes and DRG Satellite Glial Cells In Vivo

**FIGURE 2:**
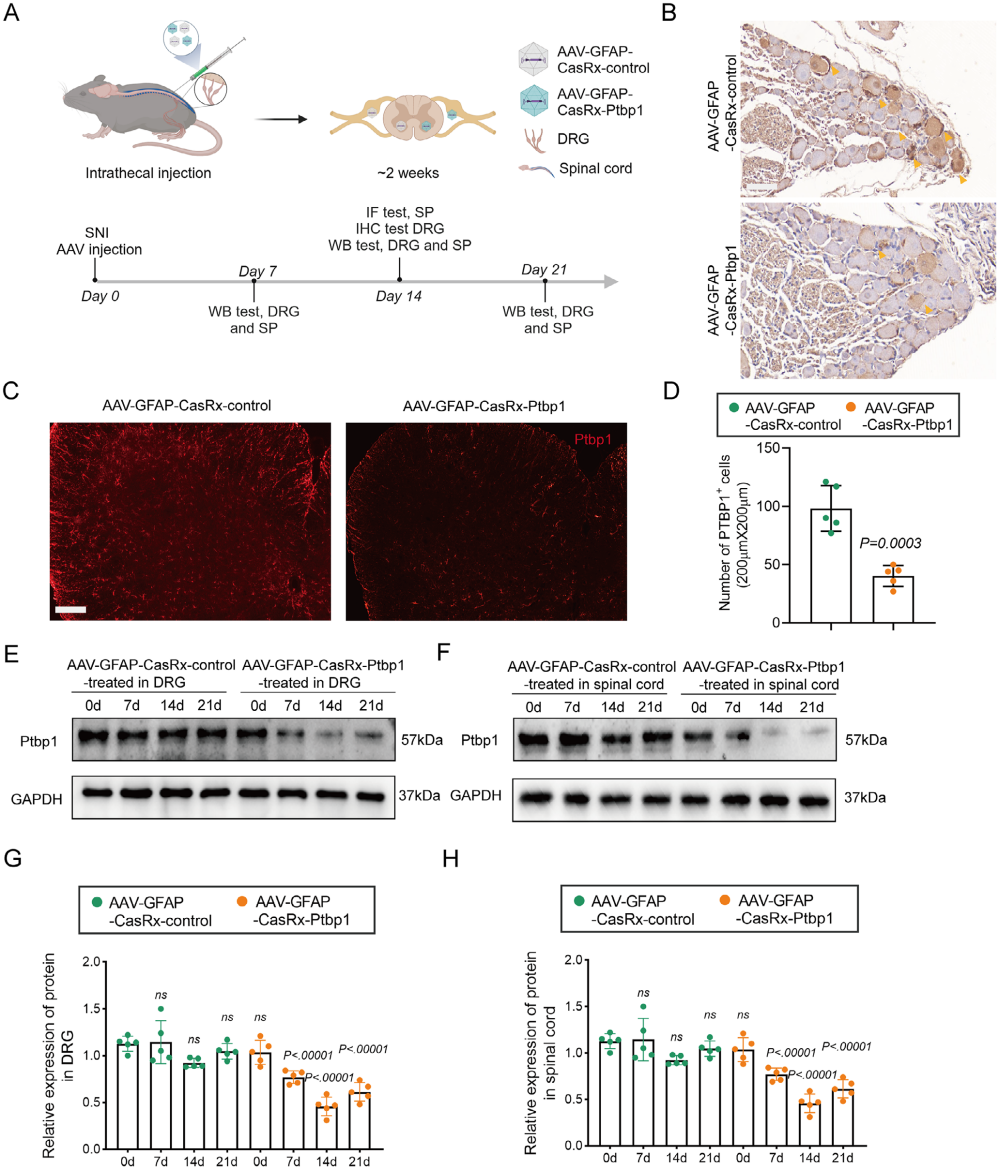
Specific knockdown of Ptbp1 in vitro using AAV. Schematic of the experiment presented in. (B) Immunohistochemistry of Ptbp1 in the DRG after AAV injection. Yellow arrowheads indicate that Ptbp1 is mainly localized in satellite glial cells. Scale bar, 100 μm. (C) Immunofluorescence of Ptbp1 in the spinal cord after AAV injection. Scale bar, 100 μm. (D) Number of Ptbp1^+^ cells after AAV injection (*n* = 5 biological replicates per group; unpaired t test; mean ± SEM). (E) Western blotting was used to measure the protein level of Ptbp1 in the DRG at different time points after AAV injection. (F) Western blotting was used to measure the protein level of Ptbp1 in the spinal cord at different time points after AAV injection. (G) Quantification of the protein level of Ptbp1 in the DRG (*n* = 5 biological replicates per group; unpaired *t* test; mean ± SEM). (H) Quantification of the protein level of Ptbp1 in the spinal cord (*n* = 5 biological replicates per group; unpaired *t* test; mean ± SEM).

To effectively target the knockdown of Ptbp1 in the spinal cord and DRG, we developed AAV‐GFAP‐CasRx‐Ptbp1 to specifically knock down Ptbp1 in astrocytes or SGCs using gRNAs targeting Ptbp1, under the control of the GFAP promoter. Additionally, we constructed AAV‐GFAP‐CasRx‐control, which lacks Ptbp1 gRNAs as previously reported [28]. AAVs were injected intrathecally because the virus could be distributed to all segments of the spinal cord and DRGs after intrathecal injection, as evidenced by existing literature [29]. On the 7th day post‐injection, immunofluorescence staining of the spinal cord and DRG confirmed successful AAV transfection. CasRx was co‐stained with GFAP, indicating effective transfection into astrocytes and satellite glial cells (Figure S2A,B). To further evaluate the outcomes, on the 14th day after injection, the spinal cords of the mice were subjected to immunofluorescence staining, and the DRG was subjected to immunohistochemistry. Ptbp1 protein expression in the spinal cord and DRG was tested on the 7th, 14th, and 21st days by WB (Figure 2A). Immunohistochemical analysis revealed that Ptbp1 expression notably reduced in the DRG (Figure 2B), while immunofluorescence staining demonstrated that Ptbp1 expression substantially decreased in the spinal cord (Figure 2C) with statistical significance (Figure 2D). Moreover, WB analysis revealed that Ptbp1 expression in both the spinal cord and DRG markedly decreased on the 14th and 21st days post‐AAV intervention (Figure 2E) with statistical significance (Figure 2H).

### 2.3 Knockdown of Ptbp1 Promotes Nerve Regeneration

**FIGURE 3:**
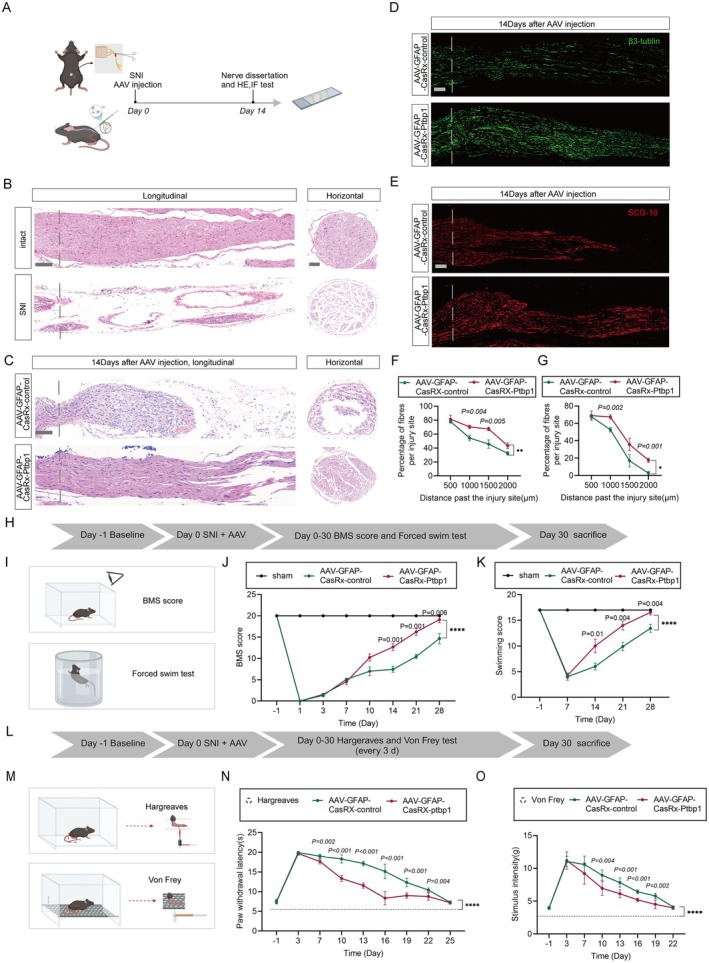
Promoting nerve regeneration and recovery of motor and sensory function. (A) Schematic of the experiment presented in. (B) HE staining for longitudinal and horizontal sections of the sciatic nerve after SNI. Scale bar, 200 μm left, 100 μm right. (C) HE staining for longitudinal and horizontal sections of the sciatic nerve on the 14th day after AAV injection. Scale bar, 200 μm left, 100 μm right. (D, E) Immunofluorescence of β3‐tubulin (D) and SCG‐10 (E) for longitudinal sections of sciatic nerves. Scale bar, 200 μm. The dashed line indicates the injury site. (F, G) Quantification of the percentage of fibers past the injury site normalized to the number of fibers at the injury site plotted as a function of the distance from the injury site (*n* = 3 biological replicates per group; two‐way‐ANOVA with Tukey's multiple comparison test; mean ± SEM; **p < 0.01, *p < 0.05). (H) Schematic of the behavior test. (I) Schematic illustration of BMS and LSS score test. (J, K) The Basso Beattie Bresnahan (BBB) locomotion scores (J) and Louisville Swim scores (K) of different groups over 30‐day period (*n* = 6 biological replicates per group; two‐way‐ANOVA with Tukey's multiple comparison test; mean ± SEM, ****p < 0.0001). (L) Schematic of the behavior test. (M) Schematic illustration of thermal nociception and mechanical allodynia. (N) Visualization of the paw withdrawal latency in seconds for the Hargreaves test for thermal pain sensation (*n* = 6 biological replicates per group; two‐way‐ANOVA with Tukey's multiple comparison test; mean ± SEM; ****p < 0.0001). (O) Von Frey analysis for nociception with stimulus intensity shown in grams (*n* = 6 biological replicates per group; two‐way‐ANOVA with Tukey's multiple comparison test; mean ± SEM).

To evaluate the effective establishment of the SNI model, the sciatic nerve injury model was assessed 2 h post‐injury, and HE staining indicated a disruption of nerve fiber continuity in the SNI group compared to the sham‐operated group (Figure 3B).

While peripheral nerves exhibit remarkable regenerative abilities compared to the central nervous system, they frequently demonstrate incomplete recovery in instances of peripheral nerve disorders or trauma [2]. To determine the effectiveness of AAV‐mediated Ptbp1 knockdown in promoting sciatic nerve injury repair in mice, sciatic nerve samples underwent HE staining and immunofluorescence staining for β3‐tubulin and SCG‐10 (Figure 3A). Mice were randomly allocated into two groups (*n* = 6) and intrathecally injected with either AAV‐GFAP‐CasRx‐control or AAV‐GFAP‐CasRx‐Ptbp1 immediately after modeling; subsequently, axonal regeneration was assessed 14 days post‐SNI. H&E staining showed that nerve repair occurred more efficiently in the AAV‐GFAP‐CasRx‐Ptbp1 group than in the control group, as evidenced by well‐aligned nerve fibers, robust axonal regeneration, and reduced inflammatory cell infiltration (Figure 3C). Axonal regeneration was substantially augmented, as demonstrated by immunofluorescence staining showing enhanced β3‐tubulin‐positive nerve fibers and SCG‐10‐positive regenerating axons, post‐administration of AAV‐GFAP‐CasRx‐Ptbp1 after injury. The axonal regeneration length in the experimental group was substantially greater than that in the control group (Figure 3D,E) with statistical significance (Figure 3F,G).

### 2.4 Knockdown of Ptbp1 Promotes Recovery of Motor and Sensory Function

We observed that knocking down Ptbp1 following SNI promotes nerve regeneration. Building on this, we investigated whether Ptbp1 knockdown enhances the recovery of motor and sensory functions, which could indirectly indicate improved nerve regeneration. Motor function recovery was evaluated using the Basso Mouse Scale (BMS) and the Louisville Swim Score (LSS), potentially driven by the re‐innervation of motor nerve fibers (Figure 3H,I). Motor function in the mice began to improve progressively from the 10th day after SNI. The AAV‐GFAP‐CasRx‐Ptbp1 group demonstrated significantly faster recovery compared to the control group (Figure 3J,K). Mice in the experimental group consistently exhibited better body balance and motor coordination. Sensory function recovery was evaluated by analyzing responses to thermal stimuli and assessing the occurrence of mechanical allodynia, potentially arising from the re‐innervation of sensory nerve fibers (Figure 3L,M). During the Hargreaves test, mice administered AAV‐GFAP‐CasRx‐Ptbp1 demonstrated a hastened recovery from thermal nociception compared to the control group, as indicated by the shortened withdrawal response time from the 7th to the 19th day (Figure 3N) with statistical significance. Similarly, the difference in mechanical allodynia between the AAV‐control and AAV‐Ptbp1‐treated groups was significant, with mice exhibiting faster recovery of mechanical nociception as assessed by the Von Frey filament test, with a statistically significant reduction in flinch response time from the 7th to the 19th day (Figure 3O). The data presented in this study suggest that the knockdown of Ptbp1 accelerates both motor and sensory functions' recovery processes.

### 2.5 Depletion of Ptbp1 Induced Astrocyte‐To‐Neuron Conversion

**FIGURE 4:**
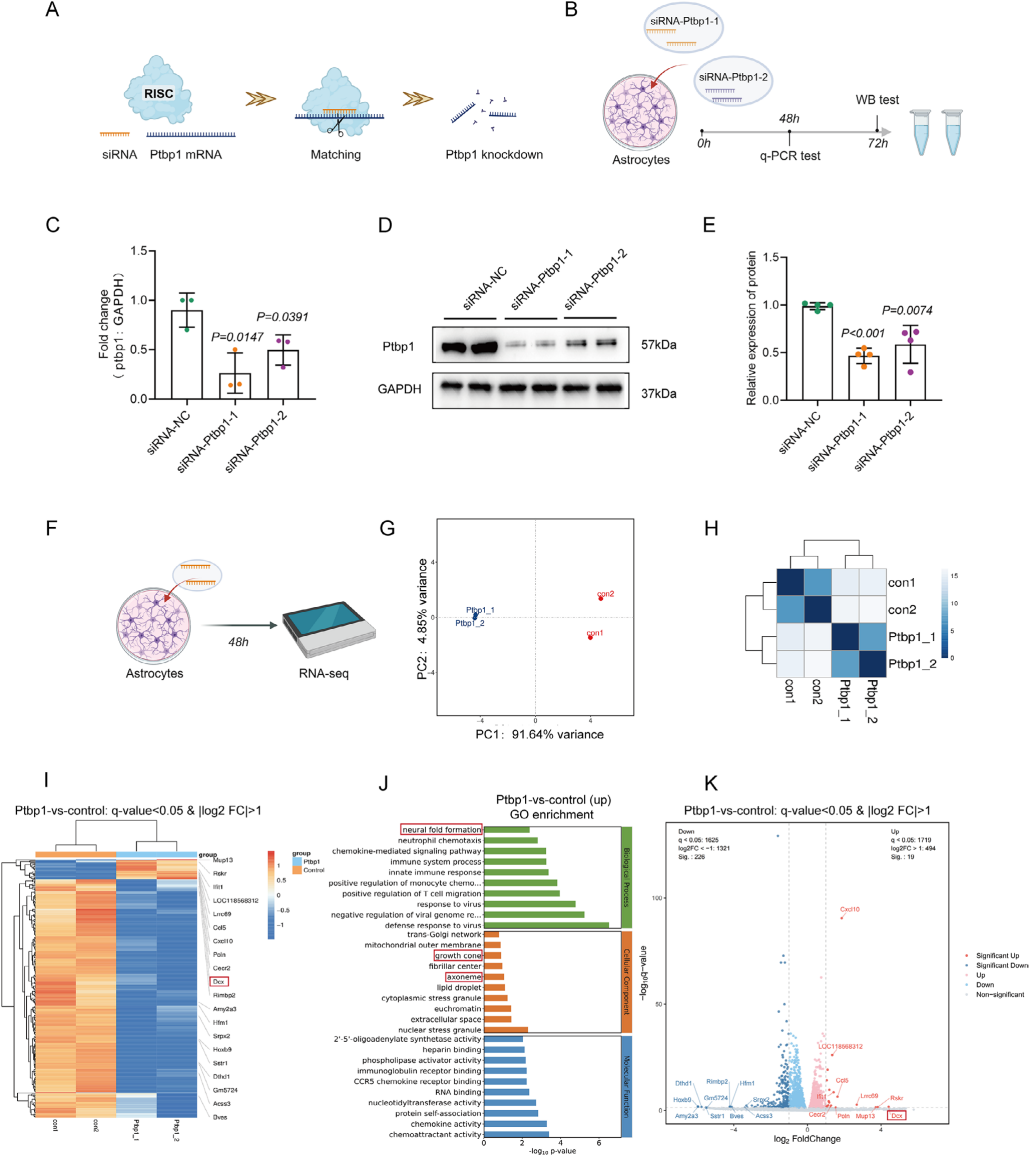
Specific knockdown of Ptbp1 in vitro using small‐interfering RNA. (A) Schematic illustration of siRNA‐mediated knockdown of Ptbp1 mRNA. (B) Schematic illustration of the test of knockdown efficiency of siRNAs. (C) Knockdown efficiency of different siRNA sequences as assessed by q‐PCR (*n* = 3 biological replicates per group; unpaired t test; mean ± SEM). (D, E) Western blotting (D) and quantification (E) were used to determine the protein level of Ptbp1 in astrocytes after siRNA intervention (*n* = 4 biological replicates per group; unpaired *t* test; mean ± SEM). (F) Schematic illustration of the follow‐up experiment. (G, H) Principal component analysis (G) and cluster analysis (H) of RNA‐seq for available samples. (I) Heatmap of differentially regulated genes. Blue: Downregulated, red: Upregulated. (J) Shown are significantly upregulated GO pathways with −log_10_ *p* < 0.05. (K) Volcano map of RNA‐seq; gray are genes with nonsignificant differences, and red and blue are significantly different genes; the horizontal axis is log_2_FoldChange, and the vertical axis is −log_10q‐value_.

**FIGURE 5:**
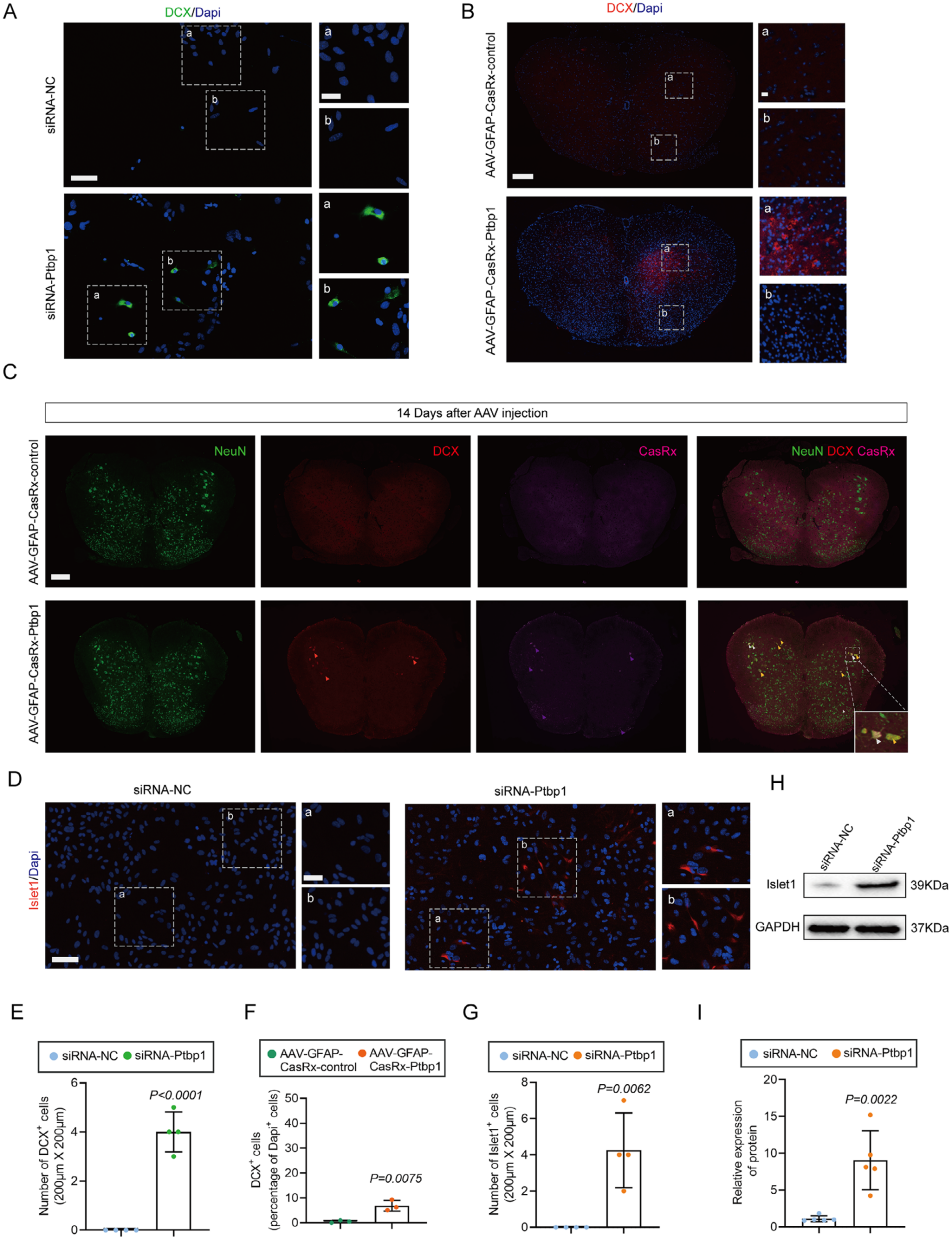
Emergence of newborn neurons in vitro and the spinal cord. (A) Immunofluorescence of DCX in vitro at 96 h after siRNA intervention. Scale bar, 50 μm (left), 20 μm (right). (B) Immunofluorescence of DCX in the spinal cord at 14 days after AAV injection. Scale bar, 200 μm (left), 20 μm (right). (C) Immunofluorescence of Neun, DCX, and CasRx in vitro at 14 days after AAV injection. Scale bar, 200 μm. (D) Immunofluorescence of Islet1 in vitro at 96 h after siRNA intervention. Scale bar, 50 μm (left), 20 μm (right). (E, F) Number of DCX^+^ cells (E) and Islet1^+^ cells (F) in vitro (*n* = 4 biological replicates per group; unpaired t test; mean ± SEM). (G) Number of DCX^+^ cells in the spinal cord (*n* = 3 biological replicates per group; unpaired *t* test; mean ± SEM). (H, I) Western blotting (H) and quantification (I) were used to determine the protein level of Islet1 after siRNA intervention (*n* = 5 biological replicates per group; unpaired *t* test; mean ± SEM).

Delayed nerve repair reduces astrocyte plasticity, creating a less supportive environment for regeneration in the peripheral nervous system. Consequently, elucidating the underlying mechanisms of astrocyte plasticity has garnered considerable interest [30]. To investigate potential alterations in astrocytes post‐Ptbp1 knockdown, we cultured primary astrocytes in vitro and confirmed their purity via immunofluorescence, ensuring the absence of neurons and neural stem cells (Figure S3A,B). Ptbp1 knockdown was achieved using small interfering RNA (siRNA) specifically targeting the gene, and cell samples were subsequently collected for comprehensive transcriptome sequencing (Figure 4A,F). Initially, to ascertain the siRNA‐mediated knockdown efficacy, we screened two distinct siRNAs and conducted a quantitative assessment of transcript and protein levels employing q‐PCR and WB analysis. The outcomes revealed that siRNA‐Ptbp1‐1, selected for further experimentation, exhibited superior efficacy compared to its alternative counterpart (Figure 4B–E). Transcriptome sequencing and cluster analysis were subsequently performed for available samples (Figure 4G,H). Remarkably, heatmap analysis of significantly differentially expressed genes (DEGs) unveiled a pronounced upregulation of DCX expression, a hallmark of newborn neurons, 48 h following siRNA intervention (Figure 4I). The volcano plot further highlighted DCX as having the highest differential fold change among the DEGs (Figure 4K). Subsequently, we evaluated DCX expression in vitro employing immunofluorescence staining, which confirmed the successful neuronal transdifferentiation post‐Ptbp1 knockdown (Figure 5A) with statistical significance (Figure 5E).

To ascertain if analogous effects could be replicated in vivo, we utilized immunofluorescence analysis to detect alterations in DCX expression consequent to AAV intervention. Remarkably, a notable elevation in DCX expression was discerned in the spinal cord of the AAV‐GFAP‐CasRx‐Ptbp1 group compared to the control group (Figure 5B) bearing statistical significance (Figure 5F), whereas no such increase was observed in the DRG, demonstrating that the knockdown of ptbp1 in satellite glial cells failed to induce cell conversion (Figure S4A,B). To confirm whether the newborn neurons were derived from AAV‐transfected astrocytes, we performed co‐staining for DCX and CasRx. The results revealed co‐localization of DCX with CasRx, indicating that the newborn neurons originated from transfected astrocytes (Figure 5C), as confirmed statistically (Figure S5).

We further observed that the newborn neurons in the spinal cord were predominantly located in the anterior horn (Figure 5B). Additionally, transcriptome analysis revealed an upregulation of the motor neuron marker following the intervention, while its expression remained undetectable in the control group. These findings suggest that knocking down Ptbp1 induces astrocyte transdifferentiation into motor neurons. To confirm this, we conducted both immunofluorescence and WB analyses to quantify Islet1 expression, discovering a substantial augmentation in Islet1 expression 48 h after the siRNA‐Ptbp1 intervention (Figure 5D,H), as confirmed statistically (Figure 5G,I).

### 2.6 Newborn Neurons Mature and Outgrow Morphologically Complete Axons

**FIGURE 6:**
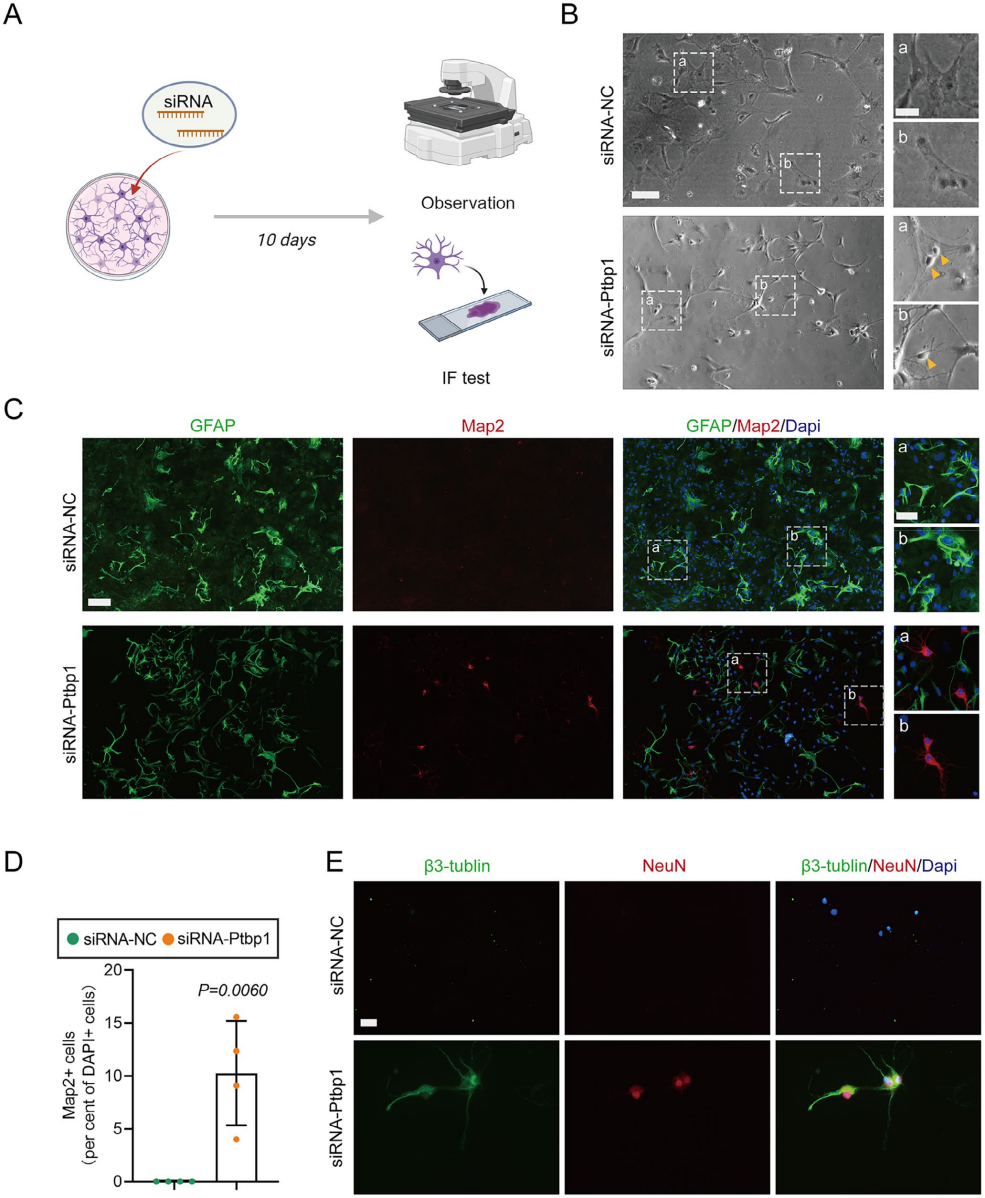
Newborn neurons mature and outgrow morphologically intact axons in vitro. (A) Schematic of the experiment presented. (B) Observation employing inverted phase‐contrast microscopy. Scale bar, 100 μm. (C) Immunofluorescence staining of Map2 with GFAP in vitro. Scale bar, 100 μm. (D) Quantification of the percentage of Map2^+^ cells 10 days after siRNA intervention (*n* = 4 biological replicates per group; unpaired t test; mean ± SEM). (E) Immunofluorescence of β3‐tubulin, which indicates that neurons gradually mature and outgrow morphologically complete axons 10 days after siRNA intervention; scale bar, 20 μm.

To verify the capacity for maturation and axonal sprouting in newly transdifferentiated neurons, we prolonged the duration of the siRNA intervention to 10 days (Figure 6A). Initially, employing inverted phase‐contrast microscopy, it was observed that neurons, characterized by small and bright nuclei, emerged within the astrocyte population, with some neurons having already established axonal connections (Figure 6B). Subsequently, immunofluorescence staining was utilized to co‐stain Map2, a widely recognized neuronal marker, and GFAP. This confirmed the maturation of newly transdifferentiated neurons within the astrocyte population post 10 days of siRNA‐Ptbp1 intervention (Figure 6C) with statistical significance (Figure 6D). To further investigate the morphological features of neuronal protrusions, we utilized immunofluorescence staining to detect the axonal marker β3‐tubulin, while concurrently co‐staining the cells with NeuN, a neuronal nucleus marker. Immunofluorescence assay results revealed that the transdifferentiated neurons effectively sprouted morphologically intact axons (Figure 6E).

### 2.7 Knockdown of Ptbp1 Promotes Astrocyte Polarization to the A2 Type and Facilitates the Production of Glial Cell‐Derived Nerve Growth Factor

**FIGURE 7:**
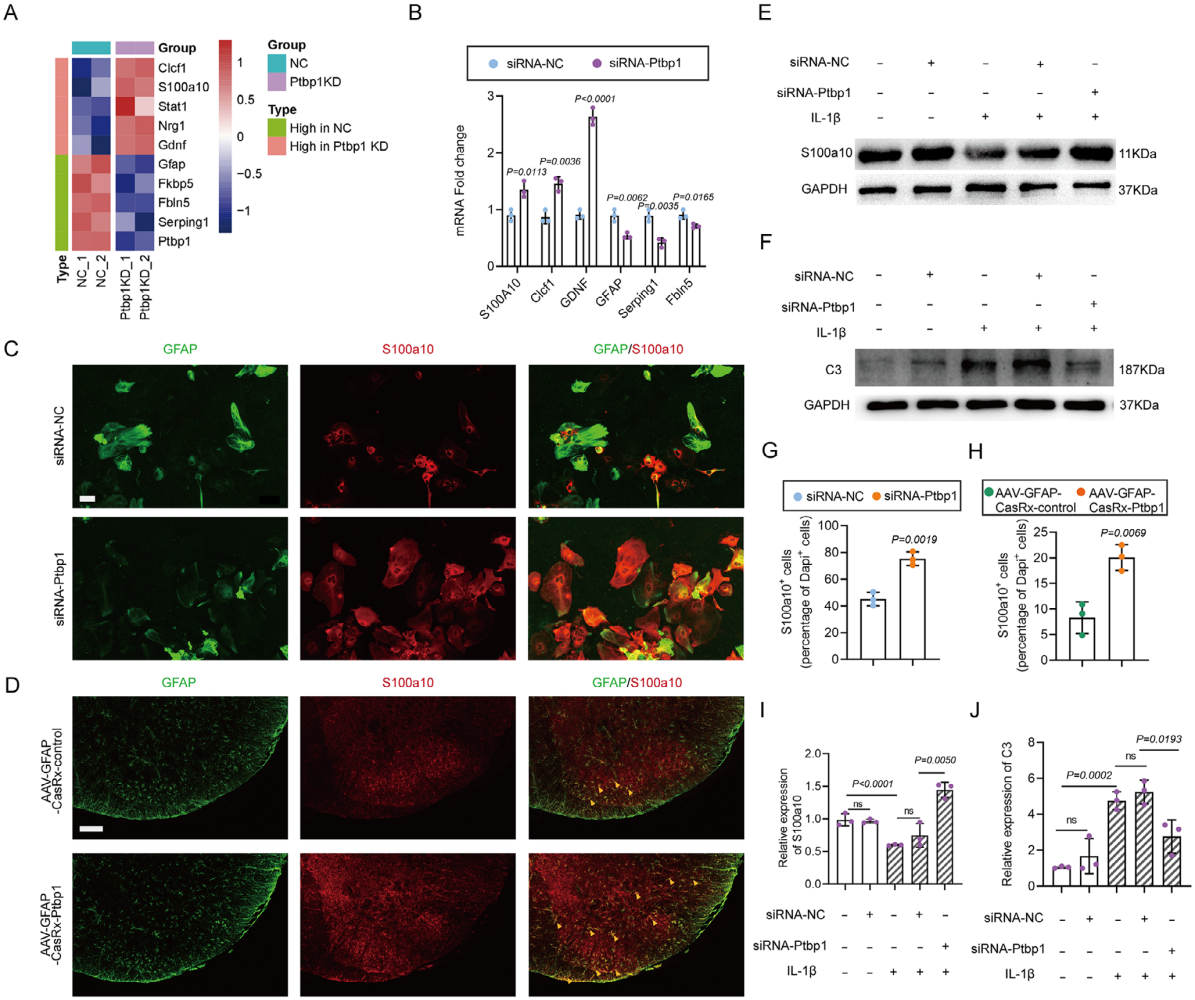
Enhancement of astrocyte polarization toward type A2 in vitro and in vivo. (A) Heatmap of the RNA‐seq data. Red indicates significantly upregulated genes, while blue indicates significantly downregulated genes. (B) Quantification of the q‐PCR results for related DEGs (*n* = 3 biological replicates per group; multiple tests; mean ± SEM). (C, D) Immunofluorescence staining of S100a10 in combination with GFAP in vitro (C) and the spinal cord (D) after knocking down Ptbp1. Scale bar, 50 μm. (E, F) Western blotting was used to measure the protein levels of S100a10 (E) and C3 (F) at 48 h after treatment with IL‐1β or siRNA. (G, H) Quantification of Western blot data for determining the protein level of S100a10 in vitro (G) and in the spinal cord (H) after knocking down Ptbp1 (*n* = 3 biological replicates per group; multiple test; mean ± SEM). (I, J) Quantification of Western blot data for determining the protein level of S100a10 (E) and C3 (F) at 48 h after treatment with IL‐1β or siRNA (*n* = 3 biological replicates per group; multiple *t* test; mean ± SEM)

Since the percentage of astrocytes converting to neurons is relatively low, we sought to investigate potential changes occurring in the remaining astrocytes. The sequencing data disclosed that astrocytes subjected to Ptbp1 knockdown manifested diverse expression patterns of numerous genes linked to astrocyte polarization. Specifically, there was an upregulation in the expression of neurotrophic A2 astrocyte markers, namely S100a10 and Clcf1, alongside a substantial elevation in GDNF expression, which is known as a potent factor of motor neuron survival and axon outgrowth [31]. Conversely, the expression of neurotoxic A1 astrocyte markers, encompassing Serpinig1, Fkbp5, and Fbln5, was notably reduced (Figure 7A). Accordingly, q‐PCR analysis was conducted to scrutinize the expression of pertinent genes post siRNA‐Ptbp1 treatment in astrocytes, and the outcomes aligned with those derived from transcriptome sequencing. WB results alongside confirmed significantly upregulation of GDNF expression levels (Figure S6A,B). Subsequently, we employed well‐established markers for A1 astrocytes (C3) and A2 astrocytes (S100a10) to determine the extent of astrocyte polarization. The expression levels of S100a10 in astrocytes were assessed 72 h post‐siRNA intervention utilizing immunofluorescence staining. The results indicated a substantial increment in the number of S100a10‐positive cells in the siRNA‐Ptbp1 group (Figure 7C) with statistical significance compared to the siRNA‐NC group (Figure 7G). Corresponding results were observed in vivo, with S100a10‐positive cells substantially increased in the AAV‐GFAP‐CasRx‐Ptbp1 group (Figure 7D), with these findings being statistically significant (Figure 7H).

Interleukin‐1 beta (IL‐1β) is known to elicit an inflammatory response within astrocytes. Primary astrocytes were meticulously isolated and cultivated in vitro, followed by the addition of IL‐1β (10 ng/mL) to the culture medium. After 48 h, the expression levels of S100a10 and C3 were quantitatively analyzed by WB. The results indicated a notable decrement in S100a10 expression and a corresponding increment in C3 expression. Subsequently, following the addition of IL‐1β, siRNA‐Ptbp1 was introduced, and after 48 h, the expression levels of both were assayed via WB. The results demonstrated that the expression level of S100a10 was substantially increased (Figure 7E) and that the level of C3 was substantially decreased after siRNA‐Ptbp1 intervention (Figure 7F) as confirmed by statistical analysis (Figure 7I,J). These findings suggest that the knockdown of Ptbp1 facilitates the polarization of astrocytes toward the type A2 phenotype and enhances the production of neurotrophic factors.

In summary, these findings highlight the mechanisms through which Ptbp1 knockdown in astrocytes promotes motor function recovery following SNI.

### 2.8 Knockdown of Ptbp1 Enhances DRG Axon Regeneration via Activating the ntng2/NGL‐2 Signaling Pathway

**FIGURE 8:**
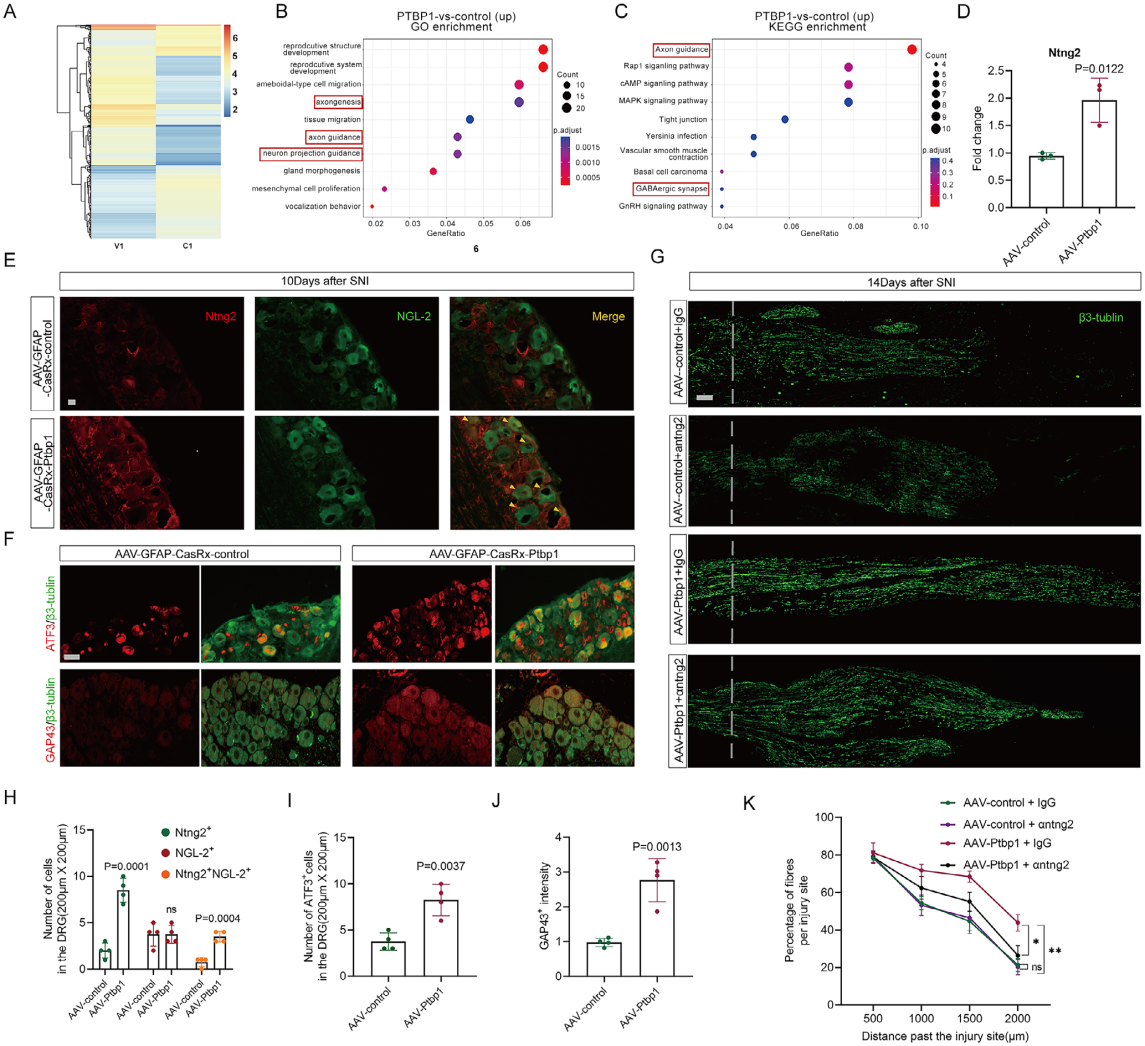
Enhancement of DRG axon regeneration via activating the ntng2/NGL‐2 signaling pathway. (A) Heatmap of the ATAC‐seq data. (B, C) Shown are significantly upregulated GO (B) and KEGG (C) pathways categorized by the Database for Annotation, Visualization, and Integrated Discovery Bioinformatics Resource with −log_10_ *p* < 0.05. (D) Quantification of the q‐PCR results for ntng2 (*n* = 3 biological replicates per group; unpaired *t* test; mean ± SEM). (E, F) Western blotting was used to measure the protein levels of S100a10 (E) and C3 (F) at 48 h after treatment with IL‐1β or siRNA. (G) Immunofluorescence of β3‐tubulin for longitudinal sections of sciatic nerves. Scale bar, 200 μm. The dashed line indicates the injury site. (H) Quantification of Ntng2, NGL‐2 cell number at 10 days after treatment (*n = 3* biological replicates per group; unpaired t‐test; mean ± SEM). (I, J) Quantification of ATF3, GAP43 cell number at 10 days after treatment (*n* = 4 biological replicates per group; unpaired *t* test; mean ± SEM). (K) Quantification of the percentage of fibers past the injury site normalized to the number of fibers at the injury site plotted as a function of the distance from the injury site (*n* = 3 biological replicates per group; two‐way‐ANOVA test; mean ± SEM; **p < 0.01, *p < 0.05)

Similar alterations were not observed in the DRG as in the spinal cord, which is likely attributable to the differential response of satellite glial cells to Ptbp1 knockdown compared to astrocytes. To explore the changes occurring in satellite glial cells after Ptbp1 knockdown, we cultured adult mouse DRG satellite glial cells in vitro. Subsequently, we performed ATAC sequencing 48 h after siRNA intervention, revealing an increase in chromatin accessibility of Ptbp1 (Figure 8A). GO enrichment analysis of upregulated genes showed significant upregulation of axonogenesis and axon guidance‐related genes (Figure 8B), while KEGG pathway enrichment analysis identified axon guidance as the most prominently enriched pathway (Figure 8C). By integrating the GO and KEGG results, we identified ntng2, an axon outgrowth‐related gene, as a potential target. Q‐PCR results also suggested that ntng2 expression increased, and the difference was statistically significant post‐Ptbp1 knockdown in vivo (Figure 8D).

The interaction between ntng2 and its specific ligand NGL‐2, known to strongly promote neuronal axon regeneration [32, 33, 34], suggests that activation of the ntng2/NGL‐2 signaling pathway may be a key mechanism. To test this hypothesis, we used immunofluorescence staining of ntng2 and NGL‐2, which revealed that ntng2 is expressed in both DRG neurons and satellite glial cells. After Ptbp1 knockdown, ntng2 expression in satellite glial cells increased significantly, and co‐staining of ntng2 and NGL‐2 was significantly enhanced in the Ptbp1‐knockdown group compared to controls, confirming activation of the ntng2/NGL‐2 signaling pathway. (Figure 8E,H). Additionally, immunofluorescence staining of axonal regeneration markers ATF3 and GAP43 confirmed a substantial enhancement of neuronal regeneration signals following Ptbp1 knockdown (Figure 8F), as confirmed by statistical analysis (Figure 8I,J).

To investigate whether activation of the ntng2/NGL‐2 signaling pathway is essential for neural regeneration after Ptbp1 knockdown, ntng2 elimination via an anti‐ntng2 monoclonal antibody following AAV‐GFAP‐CasRx‐PTBP1 intervention significantly reduced axonal regeneration after SNI compared to the IgG control group. These findings confirm that activation of the ntng2/NGL‐2 signaling pathway is required for neural regeneration under these conditions. Interestingly, axonal regeneration was not notably affected by ntng2 elimination via an anti‐ntng2 monoclonal antibody in the AAV‐control intervention group compared to the IgG control group (Figure 8G,K).

**FIGURE 9:**
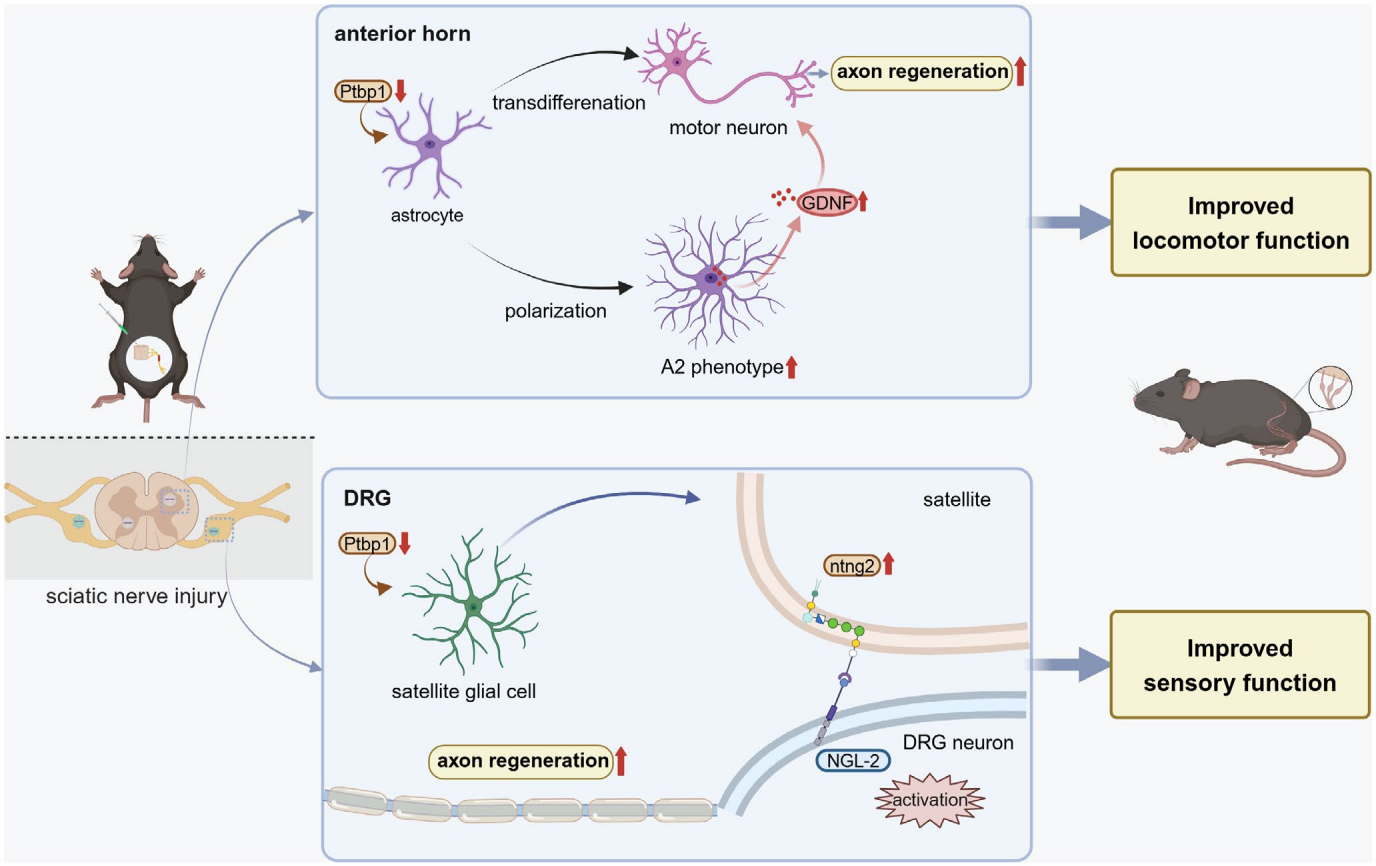
Schematic of the proposed mechanism.

Collectively, these findings demonstrate that Ptbp1 knockdown in glial cells enhances axonal regeneration and recovery of both motor and sensory functions following sciatic nerve injury (Figure 9).

## 3 Discussion

Peripheral nerve injury, a prevalent clinical ailment, substantially impairs patient quality of life, necessitating the prompt and well‐organized implementation of synergistic therapies with both anti‐inflammatory and reparative effects [35, 36]. Consequently, investigating the molecular mechanisms governing peripheral nerve regeneration is crucial for facilitating the advancement of medical therapies. The development of nervous system connectivity is an intricate process, encompassing a well‐coordinated sequence of events, notably the migration of neurons and the extension of axons [37]. While numerous studies have validated the significance of neuronal migration and axon guidance in nerve regeneration [37, 38], the role of neuronal replenishment in PNI repair has received comparatively little attention. Reports indicate that nonneuronal cells can be transdifferentiated into functional neurons to substitute those lost in diseases via in situ neuronal reprogramming, sparking considerable interest in the scientific community [39, 40]. Considering that glial cells constitute the most abundant cell type in the spinal cord, the in vivo reprogramming of endogenous cells into neurons has become a highly promising approach for nerve regeneration. Historically, research efforts have largely focused on the coordinated expression of neuronal transcription factors to induce the conversion of fibroblasts or glial cells into neurons [28, 41]. Recent studies have shown that downregulation of a single gene, Ptbp1, can lead to the transdifferentiation of astrocytes into neurons, providing new therapeutic options for neurodegenerative diseases [19, 42]. In a mouse model of Parkinson's disease, researchers successfully induced the transdifferentiation of midbrain astrocytes into dopaminergic neurons by using an AAV vector expressing shPtbp1, driven by the GFAP promoter [19]. However, these results are subject to considerable debate, as another study involving lineage tracing indicated that Ptbp1 knockdown did not lead to in vivo transformation of astrocyte‐neuron [43]. In our current study, sequencing data revealed that the knockdown of Ptbp1 in astrocytes resulted in an elevated count of DEGs related to axons and neuronal components. Specifically, the neural precursor marker DCX was markedly upregulated with the largest fold change, strongly suggesting the initiation of astrocyte‐to‐neuron transdifferentiation. Immunofluorescence staining was utilized to detect DCX expression, and the findings showed that astrocytes successfully transdifferentiate into neural precursor cells. Subsequently, we detected the motor neuron markers Islet1 and Map2, along with the axonal marker β3‐tubulin. Analyses based on these markers solidly supported the potential of these newly transdifferentiated neurons to evolve into mature neurons with fully formed axons. Remarkably, the sequencing data revealed a substantial downregulation of GFAP expression in astrocytes post Ptbp1 knockdown. This reduction in the astrocytic marker may indirectly indicate the transdifferentiation of astrocytes.

Meanwhile, prior research on the protective effect of Ptbp1 knockdown in neural injuries and neurodegenerative disorders, such as Parkinson's disease and optic nerve injury, has predominantly centered on the brain [19, 28]. However, research investigating the potential of Ptbp1 knockdown to promote the repair of peripheral nerve injuries remains relatively scarce. This phenomenon may be attributed to differential effects between Ptbp1 knockout and knockdown models. Mechanistically, Ptbp1 downregulation has been shown to induce its neuronal paralog Ptbp2 in most cell types, while dual depletion of both Ptbp1 and Ptbp2 triggers cell death. In Ptbp1 knockdown cells, Ptbp2 may retain dynamic regulatory capacity [44]. However, in Ptbp1 knockout cells, induced Ptbp2 may be constrained to sustained high levels to ensure cellular viability, thereby preventing subsequent downregulation required for neuronal reprogramming [45]. Under this rationale, our experimental strategy employing Ptbp1 knockdown in mice provides mechanistic support for the observed neuronal transdifferentiation. On the basis of the findings of our current study, we report, for the first time, that Ptbp1 knockdown facilitates the repair of sciatic nerve injuries, as evidenced by HE staining and SCG‐10 and β3‐tubulin immunofluorescence analyses. Additionally, we found that this approach accelerated the recovery of thermal and mechanical pain thresholds in mice, indirectly reflecting the re‐extension and innervation of nerve fibers.

Acute trauma to the sciatic nerve initiates an inflammatory response in the distal lumbar spinal cord, resulting in the subsequent polarization of astrocytes and concurrent alterations in the neuronal microenvironment, which can hinder axonal regeneration [46, 47]. The astrocyte‐engineered microenvironment, pivotal for neurogenesis and axonal growth, is of paramount significance. Astrocytes are capable of polarizing toward either type A1 or type A2 phenotypes. Classically activated A1 astrocytes release proinflammatory mediators, leading to neurotoxic effects. Conversely, the alternative activation of A2 astrocytes leads to the secretion of anti‐inflammatory mediators and the release of diverse neurotrophic factors, thereby manifesting neuroprotective and axonal growth‐promoting properties [48]. Increasing evidence supports the significant role of astrocytes in modulating the microenvironment during brain injuries and neurological diseases [49, 50]. A study showed that NeuroD1 attenuates subarachnoid hemorrhage by attenuating reactive astrocyte‐mediated neuroinflammation and promoting astrocyte polarization to the A2 type [49]. Consequently, we further investigated whether the majority of the remaining astrocytes, aside from those undergoing successful transdifferentiation, experienced beneficial alterations. In our research, it was noted that Ptbp1 knockdown substantially increased the prevalence of type A2 astrocytes, evident both in vitro and in the spinal cord. Furthermore, this therapeutic approach resulted in an upsurge in GDNF production, consequently fostering neurogenesis post‐nerve injury and greatly enhancing neurological recuperation.

The DRG is pivotal in sensory nerve regeneration, serving as a key center for axonal regrowth and signaling [25]. Satellite glial cells within the DRG contribute to neuronal repair by undergoing phenotypic changes and interacting with neurons [26, 51]. Recent studies highlight the potential of targeting specific molecular pathways to enhance nerve repair. The ntng2/NGL‐2 signaling pathway, known for its role in axon guidance and regeneration, has garnered attention for its ability to promote sensory axon repair [32, 33, 34]. In this study, it was revealed that Ptbp1 knockdown upregulated ntng2 expression in satellite glial cells, leading to the activation of the ntng2/NGL‐2 signaling pathway, which promoted sensory axon regeneration, as evidenced by increased axonal outgrowth markers and improved sensory functional recovery in our model.

Our findings help to elucidate the role of Ptbp1 knockdown in nerve injury and regeneration and highlight the potential therapeutic value of targeting Ptbp1 in the treatment of nerve injury. However, the conversion of astrocytes to neurons in the spinal cord was relatively limited, with a subset of astrocytes undergoing A2‐type polarization, which we hypothesize may be linked to variations in the degree of Ptbp1 knockdown. Similarly, no transdifferentiation potential was detected in the DRG; this absence may stem from inherent cellular restrictions or technical limitations of our current approaches, necessitating further investigation to clarify the underlying mechanisms. Further research is required to comprehensively understand the molecular mechanisms underlying nerve injury and repair. Key areas for investigation include understanding the intrinsic mechanisms by which Ptbp1 knockdown drives the astrocyte‐to‐neuron transition, the molecular pathways involved in astrocyte polarization, and the downstream signaling pathways in DRG neurons following NGL‐2 activation. Additionally, since the preclinical model used was mice, and the regeneration ability of peripheral nerves in mice is significantly stronger than that in humans, our results may overestimate the effect of Ptbp1 knockdown in humans [52]. The experiments used an acute injury model, while human PNI is often chronic or mixed, and the scar tissue or metabolic abnormalities in the microenvironment may weaken the effect of Ptbp1 intervention. In conclusion, our findings may have potential significance for the treatment of human PNI, as the regeneration ability of peripheral nerves in humans is limited, and Ptbp1 is highly conserved in mammals, potentially making it a cross‐species intervention target.

By addressing these limitations, future research can refine the therapeutic potential of Ptbp1 targeting and advance our understanding of peripheral nerve repair mechanisms. Despite these challenges, our findings in this study offer profound insights into the intricate molecular mechanisms underpinning nerve injury and regeneration and propose that knocking down Ptbp1 represents a promising avenue for devising innovative therapeutic strategies targeting peripheral nerve injuries.

## 4 Experimental Model and Subject Details

### 4.1 Animals

Eight‐week‐old male C57BL/6J mice were sourced from the Animal Experiment Center at Naval Medical University, located in Shanghai, China. These animals were housed and underwent surgical interventions in a specific pathogen‐free (SPF) facility, adhering to the rigorous standards required for scientific publications. The mice were accommodated in cages featuring a closed‐circuit ventilation system, with no more than five mice per cage to ensure adequate space and minimize stress. Environmental conditions were strictly controlled, with the temperature set at 23°C ± 1°C, relative humidity at 55%, and a regulated 12‐h light–dark cycle to mimic natural conditions. The mice were provided with unrestricted access to standard laboratory food and water. All procedures involving animals were reviewed and approved by the Scientific Investigation Committee of NMU and were performed in strict compliance with the ethical guidelines established by the International Pain Research Association's Ethics Committee.

### 4.2 Culture of Primary Astrocytes

Astrocytes were separated from the cerebral cortex of newborn (1–2 days old) C57BL/6 mice. The cortex was gently dissected in PBS, minced into small pieces, and then treated with 1 mL of 0.25% trypsin–EDTA solution at 37°C for 15 min. After cell dissociation, the cells were centrifuged, resuspended, and plated in a T25 culture flask. Non‐astrocytic cells were removed by gently shaking the flasks daily.

### 4.3 Satellite Glial Cell Culture

SGCs were isolated from adult mice (8–12 weeks old) using a stepwise protocol to ensure high purity and integrity as previously described [53]. Following anesthesia and laminectomy, dorsal root ganglia (DRGs) were carefully dissected and transferred to a chilled culture dish containing HBSS to preserve tissue viability. The DRGs were subjected to enzymatic dissociation by incubating with papain solution (40 U/mL) at 37°C for 20 min, followed by centrifugation and rinsing with HBSS. Collagenase (37°C for 20 min) was then applied to further dissociate the tissue, and gentle pipetting was used to break up the cells. The cell suspension was filtered through 40 μm and 10 μm strainers to eliminate larger fragments and non‐target cells. The dissociated cells were planted onto a 12‐well plate with sterilized cover glasses and cultured in pre‐warmed DMEM supplemented with 10% fetal bovine serum and 1% penicillin–streptomycin. After 24 h, the medium was refreshed to remove non‐adherent material. Cells were maintained with medium changes every 2–3 days, achieving confluency within 10–14 days.

## 5 Method Details

### 5.1 Mouse Model of SNI

To ensure adequate anesthesia during the surgical procedure, mice were administered pentobarbital at a dose of 5 mg/kg. Once anesthetized, the animals were positioned prone on surgical plates, with all limbs securely taped to maintain stability. A midline incision was made in the skin overlying the dorsal thigh region. The sciatic nerve was carefully exposed by dissecting between the biceps femoris and semimembranosus muscles, near the margin of the gluteus maximus. The sciatic nerve underwent a clamping procedure using a vascular clamp for 5 s, followed by a release period of 10 s. This process was repeated, with the clamp applied at a 2 mm distance from the original site, for a total of three cycles. Noninvasive clamps were utilized to demarcate the proximal boundary of the injury site. Following the completion of the procedure, the skin incision was meticulously closed using #7 surgical sutures.

### 5.2 Intrathecal Injection in Mice

Mice were anesthetized by intrathecal injection. The mice were immobilized in the prone position on a mouse immobilization frame, and the pelvic bones of the mouse were grasped with the thumb and index finger of the nondominant hand, with the top of the hand resting gently on the body and head of the mouse. While holding the mouse gently upward with the nondominant hand to open the intervertebral space, the dominant hand used the index finger to gently press along the spine from the lumbar to the sacral region. The iliac spine, which connects both sides, corresponds to the sixth lumbar vertebra. The needle, with its bevel facing the mouse's head, was inserted into the midline of the lumbar vertebrae at a 70° angle. Upon feeling the needle touch the bone, the angle was adjusted to a 30° pinch angle, and the needle was inserted between the L5 and L6 vertebral segments. Subsequently, 5 μL of the AAV viral suspension was injected into the subarachnoid space. The needle was then left in place for 30 s before being withdrawn.

### 5.3 Mechanical Allodynia

In the animal behavior test room, the animals were first placed in a clear plastic box and allowed to adapt to a wire mesh platform for 30 min. Using the electronic von Frey test (IITC/Life Science, USA) and the proper probe stimulating the plantar sole of the hind paws with increasing force, the paw mechanical withdrawal threshold (PWT) was determined. If an external stimulus, such as licking, muscle contraction, or running away, triggered a paw withdrawal reaction, this stimulus was logged in the computer. Each animal underwent three measurements 10 min apart, and the PWT was defined as the mean of the three repeated tests. The PWT was measured every 3 days for 30 days after AAV injection. The baseline value was determined 1 day before the model was created.

### 5.4 Thermal Nociception

The thermal withdrawal latency (TWL) was measured after the mice were kept on a glass surface in a transparent plastic box under the same conditions for 30 min. To elicit paw withdrawal responses, the hind paw was stimulated with a Hargreaves radiant heat device (IITC/Life Science, USA). To prevent tissue damage, a limit of 20 s was chosen. The TWL was determined by three measurements taken 10 min apart for each animal, after which the results of the three duplicate tests were averaged. TWL was measured every 3 days for 30 days after AAV injection. The base value of the TWL was determined 1 day before the model was created.

### 5.5 Motor Function Evaluation

Hindlimb motor function was assessed using two validated scoring systems: the Basso Mouse Scale (BMS) and the Louisville Swim Score (LSS), as outlined in previous research [54]. Initially, mice were given a 5‐min acclimation period in the open field. Following this, two trained and independent evaluators, who were blinded to the experimental conditions, scored hindlimb functionality using the BMS. This scale quantifies motor recovery on a range from 0 (indicating total paralysis) to 21 (representing normal locomotion), focusing on joint mobility and coordination during movement. Evaluations were performed pre‐operatively and at post‐injury intervals of days 1, 3, 7, 10, 14, 21, and 28, with six mice in each group.

In parallel, the LSS, which operates on a 0–15 scale, was employed to evaluate parameters such as forelimb reliance, hindlimb synchronization, body posture, and trunk control during aquatic activity. Mice were initially familiarized with the aquatic environment and trained to navigate a water‐filled glass tank. Each mouse was evaluated twice: once before surgery and weekly thereafter for up to 1 month.

### 5.6 Astrocyte Transdifferentiation In Vitro

To initiate transdifferentiation under in vitro conditions, astrocytes were seeded into six‐well culture plates and maintained in astrocyte growth medium. This medium consisted of DMEM/F12 (GIBCO), supplemented with 10% fetal bovine serum (FBS, GIBCO) and penicillin/streptomycin (GIBCO). Upon reaching 70%–80% confluency, the cells were transfected with small interfering RNA (siRNA) specifically targeting Ptbp1. SiRNA sequences have been addressed in Table S1. Following a 48‐h incubation period, the culture medium was replaced with a 1:1 mixture of DMEM/F12 and neurobasal medium, enriched with N3 supplements, 25 μg/mL insulin, 0.4% B27, and 2% FBS. The medium was partially refreshed every 2 days to maintain optimal culture conditions.

### 5.7 Western Blot Analysis

Spinal cord and DRG tissues or treated astrocytes were lysed in RIPA lysis buffer (Epizyme, China) containing a protease and phosphate inhibitor. A BCA protein assay kit was used to measure the protein concentrations. Equal volumes of protein (30 μg) were electrophoretically transferred to 0‐point 2‐μm polyvinylidene fluoride (PVDF) membranes (Millipore, USA) after separation via 10% or 12‐point 5% SDS–PAGE. The membrane was incubated with a protein‐free rapid‐blocking buffer (Epizyme, China) for 1 h at room temperature under constant agitation on a rocker. This was followed by overnight incubation at 4°C with specific primary antibodies, such as anti‐GAPDH (1:20,000), anti‐Ptbp1 (1:1000), anti‐C3 (1:1000), anti‐S100A10 (1:1000), and anti‐Islet1 (1:1000), following with the treatment of membranes with HRP‐conjugated secondary antibodies The presence of target proteins was subsequently quantified using an enhanced chemiluminescence detection system. The relative expression of the target protein was determined by dividing the expression level of the target protein by that of GAPDH. The densitometric analysis of the protein bands was performed using ImageJ software to ensure accurate quantification of signal intensities.

### 5.8 Quantitative Real‐Time Polymerase Chain Reaction

Total RNA was isolated using RNAfast200 reagent, followed by reverse transcription into complementary DNA (cDNA) with the PrimeScript RT Reagent Kit, which includes a gDNA Eraser (TaKaRa RR037A), adhering to the manufacturer's instructions. Primer sequences were designed based on data obtained from the PrimerBank database. Quantitative reverse transcription PCR (qRT–PCR) was conducted using SYBR Premix Ex Taq (TaKaRa RR420A) on an Applied Biosystems ABI 7900HT fast real‐time PCR system. The relative mRNA expression levels were normalized to the housekeeping gene GAPDH, and the 2(−ΔCt) method was applied to quantify the relative expression levels of the target genes.

### 5.9 Immunofluorescence Staining

To stain spinal cord sections, experimental mice were euthanized with carbon dioxide and subjected to perfusion with 15–20 mL of saline (0.9% NaCl), followed by 15 mL of 4% PFA in PBS to fix the tissue. The cervical vertebrae were removed, and both sides of the spinal canal were dissected to expose the spinal cord. Subsequently, the entire region of lumbar enlargement tissue was removed and placed in 4% PFA solution, followed by storage at 4°C for 4–6 h postfixation. Following postfixation, the lumbar enlargement tissues were subjected to dehydration in 10%, 20%, and 30% sucrose solution and subsequently preserved in a refrigerator at 4°C for sectioning and further experimentation. Next, the lumbar enlargement tissue was cut into 20 μm slices, washed with CB solution, and heated in a water bath at 95°C for 10 min to restore antigen. Immunofluorescence staining was performed as described previously with anti‐Ptbp1 (1:500), anti‐DCX (1:200), anti‐S100A10 (1:200), anti‐NeuN (1:1000), anti‐Iba1 (1:1000), anti‐GFAP (1:1000), anti‐β3‐tubulin (1:500), anti‐SCG‐10 (1:500), anti‐Islet1 (1:1000), anti‐Cas13d (1:200), and anti‐ntng2 (1:500) primary antibodies. The secondary antibody (FITC‐goat anti‐mouse IgG, 1:400) was diluted in PBS, incubated for 1 h at room temperature, and washed with PBST buffer, after which 50% glycerol was added. The slide was subsequently sealed and covered. Fluorescence signals were then visualized using a fluorescence microscope (IX71; Olympus Corporation, Tokyo, Japan). Fluorescence intensity was quantified by subtracting the background fluorescence, and the average intensity for each pixel was calculated. For cultured cells, the slides were dipped three times in PBS and then fixed in 4% paraformaldehyde for 15 min. The slides were then soaked three times in PBS and permeabilized with 0.5% Triton X‐100 in PBS for 20 min at room temperature. The slides were then washed three times with PBS for 3 min each. The sealing solution was absorbed onto blotter paper for 30 min without washing, after which an adequate amount of diluted primary antibodies, as mentioned previously, was added to each slide. The slides were then placed in a humid chamber and incubated overnight at 4°C. The remaining steps were performed as described for the spinal cord sections.

## 6 Quantification and Statistical Analysis

The data are expressed as mean ± standard deviation (SD). Statistical evaluations were performed using GraphPad Prism 8 software. Normality of continuous variables was assessed using the Shapiro–Wilk test. All variables met the assumption of normality (*p* > 0.05), and parametric tests were subsequently applied. Data that do not exhibit a normal/Gaussian distribution should be analyzed via a non‐parametric equivalent. For datasets following a normal distribution, comparisons between two groups were analyzed using a two‐tailed, unpaired Student's *t* test. When comparing more than two groups, one‐way analysis of variance (ANOVA) was utilized, with Dunnett's post hoc test applied for multiple comparisons where applicable. For experiments involving two independent variables, two‐way ANOVA was conducted, followed by Tukey's multiple comparison tests to identify specific differences. A significance threshold of *p* < 0.05 was set. All data analyses were performed by researchers blinded to the experimental groups, and data were derived from two to three independent experiments.

## Author Contributions

H.Y. conceived the study, designed experiments, and supervised the whole study. M.Y., Q.C., and H.S. performed the research. H.S. and L.P. performed animal experiments. H.S., D.C., and X.F. performed experiments in vitro. T.H., R.D., and H.S. performed immunostaining, western blot, and behavior tests. H.S. and H.W. analyzed data and drafted the manuscript. Q.C. and M.D. contributed to the interpretation of data and critical revision of the manuscript. All the authors have read and approved the final manuscript.

## Ethics Statement

The animal study was reviewed and approved by the Institutional Animal Care and Use Ethics Committee of Naval Medical University.

## Conflicts of Interest

The authors declare no conflicts of interest.

## Supporting information


**Figure S1.** Cellular localization of Ptbp1.

**Figure S2.** AAV transfection identification. (A) Immunofluorescence staining of CasRx in combination with GFAP in the spinal cord. Scale bar, 200 μm. (B) Immunofluorescence staining of CasRx in combination with GFAP in the DRG. Scale bar, 100 μm.

**Figure S3.** Astrocyte purity characterization. (A) Immunofluorescence staining of GFAP, Iba‐1, DCX, Nestin, MAP2 in vitro. The scale bar is shown in the figure. (B) Quantification of positive cell number (*n* = 3 biological replicates per group; multiple test; mean ± SEM).

**Figure S4.** Failed transdifferentiation in the DRG. Immunofluorescence staining of Neun in combination with DCX in the DRG. Scale bar, 100 μm. (B) Quantification of DCX^+^ cell number (*n* = 3 biological replicates per group; unpaired t test; mean ± SEM).

**Figure S5.** Quantification of DCX+/CasRx+ cells.

**Figure S6.** Upregulation of GDNF expression post Ptbp1 knockdown. (A, B) Western blotting (A) and quantification (B) were used to determine the protein level of Islet1 after siRNA intervention (*n* = 3 biological replicates per group; unpaired *t* test; mean ± SEM).

Table S1.

Table S2.

## Data Availability

The original contributions presented in the study are included in the article/Supporting Information; further inquiries can be directed to the corresponding author. This study did not generate new unique reagents. The data presented in this paper can be obtained from the lead contact upon request. All antibodies, primers, software, etc. used in the article have been addressed in Table S2. RNA‐seq data and ATAC‐seq data are accessible publicly via GEO repositories as of the publication date, with accession numbers provided in the key resources table. No original code is included in this study. Any additional information necessary to reanalyze the data is also available from the lead contact upon request.
