# Supplementary figures and images for: Ptbp1 Knockdown in Glial Cells Promotes Motor and Sensory Function Recovery After Peripheral Nerve Injury
Source: CNS Neurosci Ther. 2025 Jul 23;31(7):e70531. doi: 10.1111/cns.70531 (PMC12287381; doi:10.1111/cns.70531)

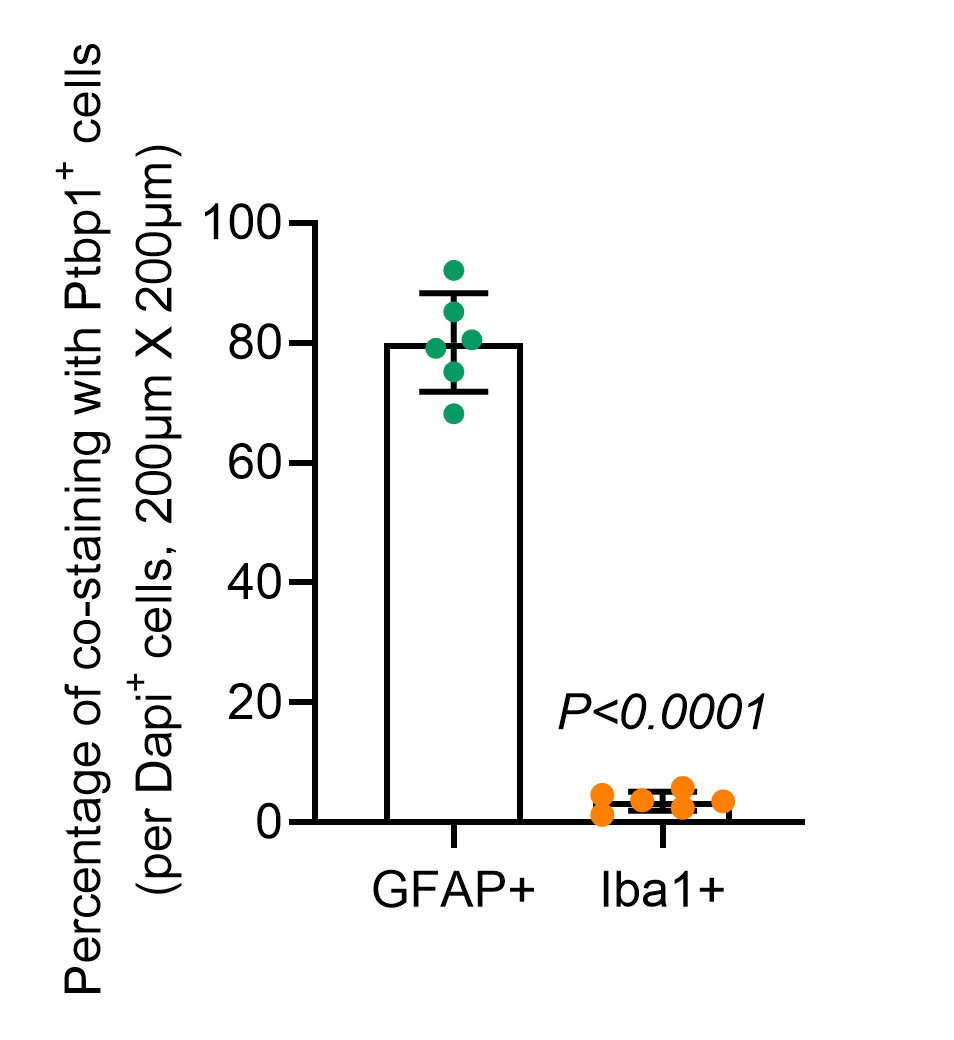

Supplement: Supplementary file 1 — Figure S1. Cellular localization of Ptbp1. [file CNS-31-e70531-s008.tif]

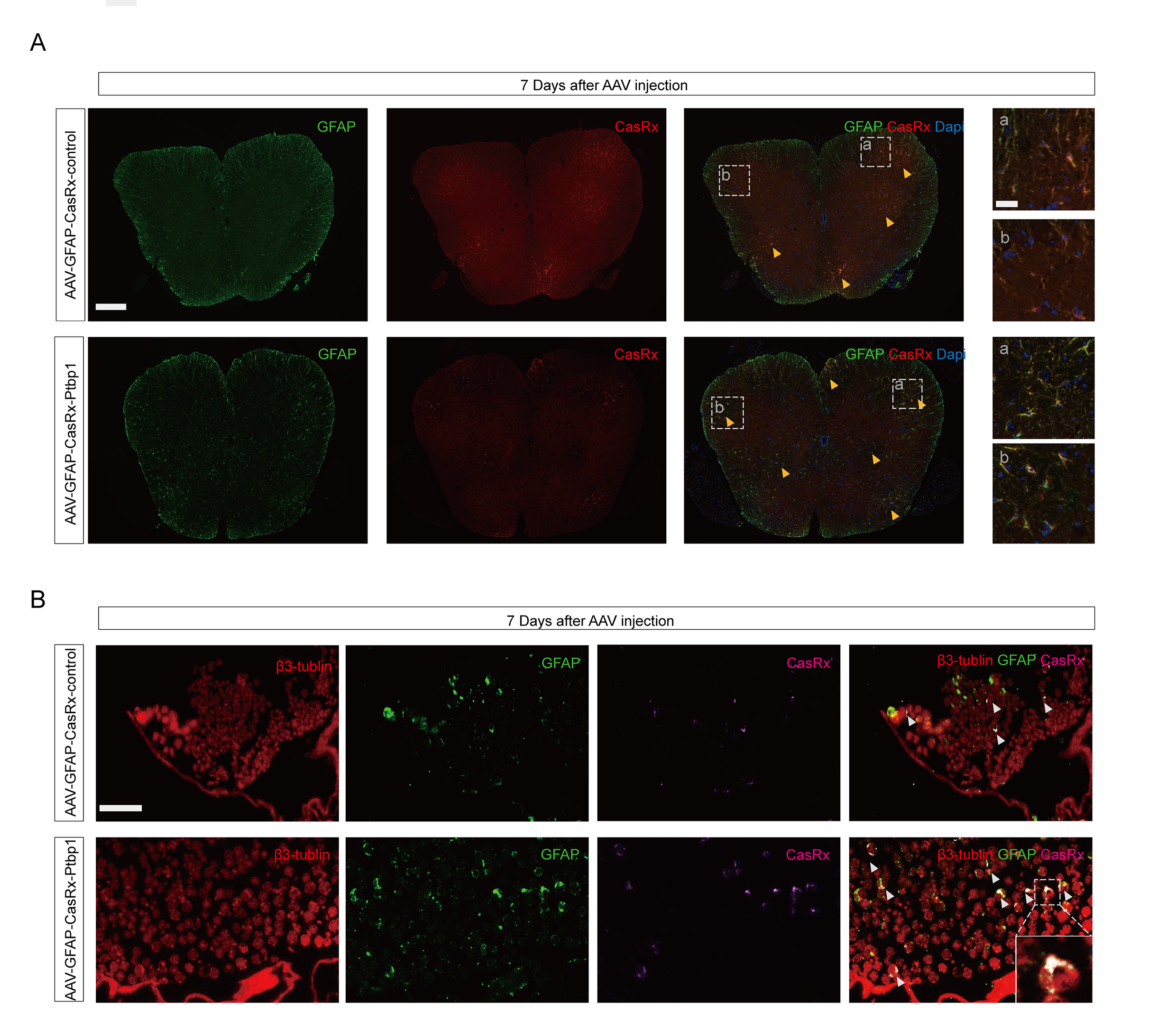

Supplement: Supplementary file 2 — Figure S2. AAV transfection identification. (A) Immunofluorescence staining of CasRx in combination with GFAP in the spinal cord. Scale bar, 200 μm. (B) Immunofluorescence staining of CasRx in combination with GFAP in the DRG. Scale bar, 100 μm. [file CNS-31-e70531-s005.tif]

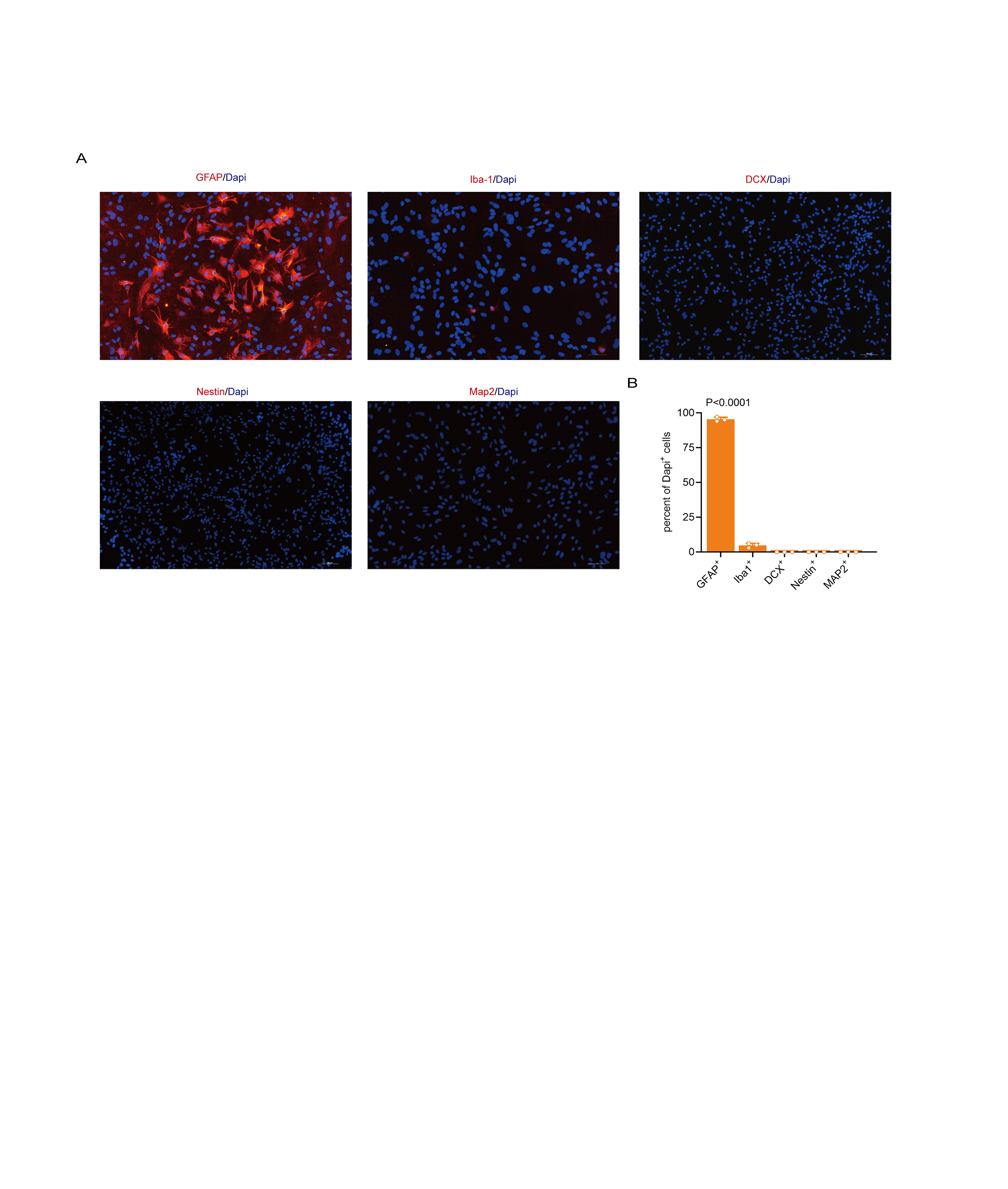

Supplement: Supplementary file 3 — Figure S3. Astrocyte purity characterization. (A) Immunofluorescence staining of GFAP, Iba‐1, DCX, Nestin, MAP2 in vitro. The scale bar is shown in the figure. (B) Quantification of positive cell number (n = 3 biological replicates per group; multiple test; mean ± SEM). [file CNS-31-e70531-s004.tif]

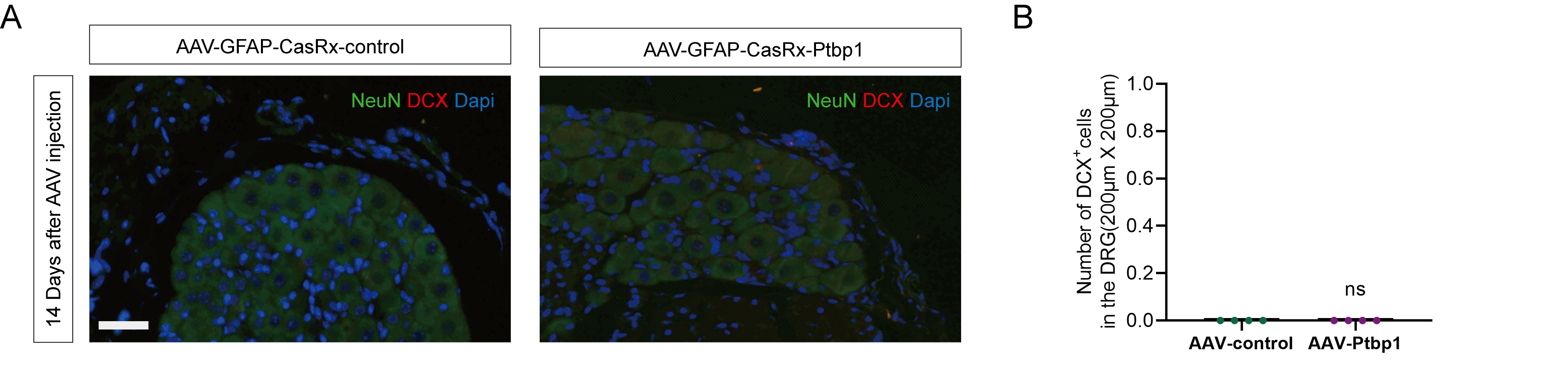

Supplement: Supplementary file 4 — Figure S4. Failed transdifferentiation in the DRG. Immunofluorescence staining of Neun in combination with DCX in the DRG. Scale bar, 100 μm. (B) Quantification of DCX+ cell number (n = 3 biological replicates per group; unpaired t test; mean ± SEM). [file CNS-31-e70531-s001.tif]

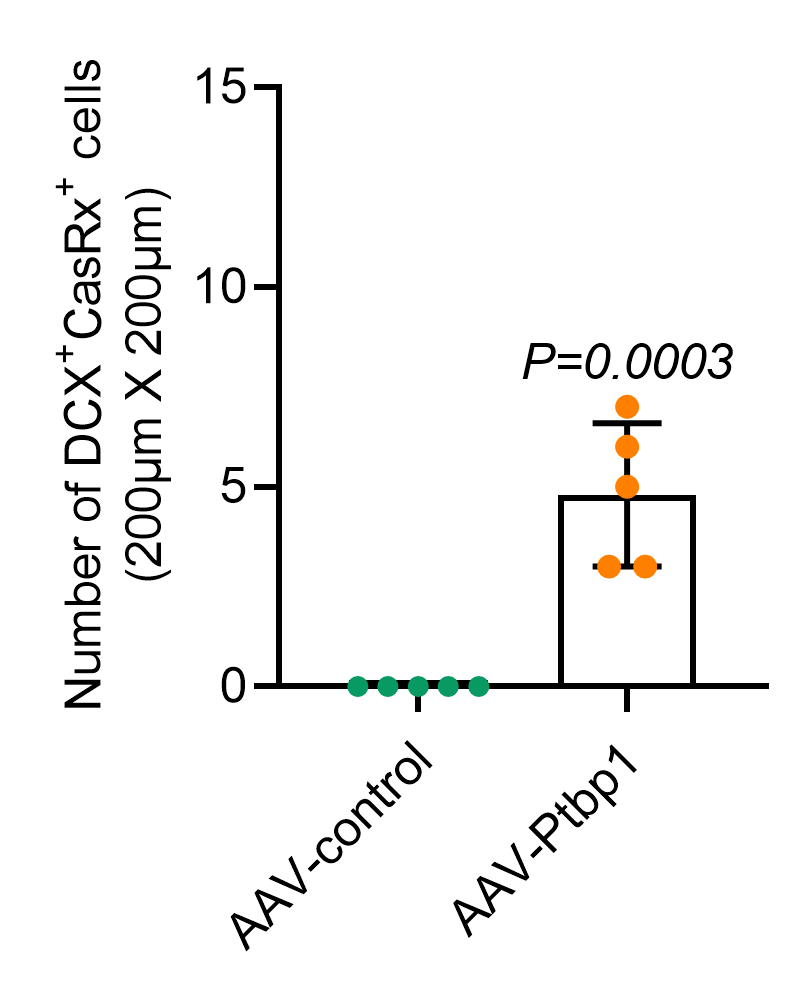

Supplement: Supplementary file 5 — Figure S5. Quantification of DCX+/CasRx+ cells. [file CNS-31-e70531-s002.tif]

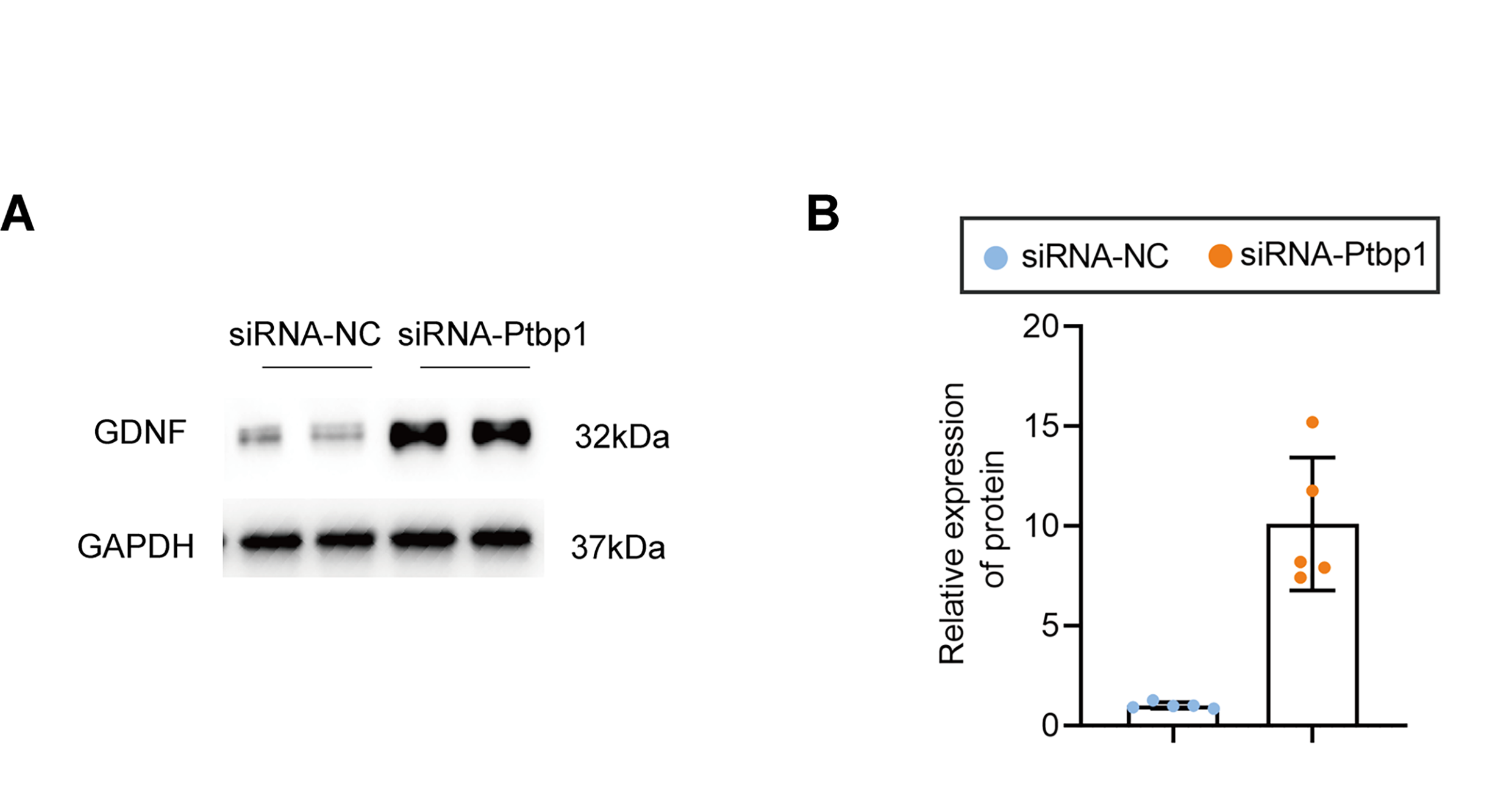

Supplement: Supplementary file 6 — Figure S6. Upregulation of GDNF expression post Ptbp1 knockdown. (A, B) Western blotting (A) and quantification (B) were used to determine the protein level of Islet1 after siRNA intervention (n = 3 biological replicates per group; unpaired t test; mean ± SEM). [file CNS-31-e70531-s006.tif]
