# Supplementary material for: Ptbp1 Knockdown in Glial Cells Promotes Motor and Sensory Function Recovery After Peripheral Nerve Injury
Source: CNS Neurosci Ther. 2025 Jul 23;31(7):e70531. doi: 10.1111/cns.70531 (PMC12287381; doi:10.1111/cns.70531)
Supplement: Supplementary file 7 — Table S1. [file CNS-31-e70531-s003.docx]

**Supplementary Table 1**. Target sequences of siRNAs

| No. | siRNA | Interference target |
| --- | --- | --- |
| 1 | siRNA-Negative control (NC) | sense: 5’- UUCUCCGAACGUGUCACGUTT-3’  antisense: 5’- ACGUGACACGUUCGGAGAATT-3’ |
| 2 | siRNA-Ptbp1-1 | sense: 5’- GCUGCCAACACUAUGGUUATT-3’  antisense: 5’- UAACCAUAGUGUUGGCAGCTT-3’ |
| 3 | siRNA-Ptbp1-2 | sense: 5’- CCCAAAGCCUCUUUAUUCUTT-3’  antisense: 5’- AGAAUAAAGAGGCUUUGGGTT-3’ |
