# Supplementary material for: Ptbp1 Knockdown in Glial Cells Promotes Motor and Sensory Function Recovery After Peripheral Nerve Injury
Source: CNS Neurosci Ther. 2025 Jul 23;31(7):e70531. doi: 10.1111/cns.70531 (PMC12287381; doi:10.1111/cns.70531)
Supplement: Supplementary file 8 — Table S2. [file CNS-31-e70531-s007.docx]

**Supplementary table 2: Key resources table**

| REAGENT or RESOURCE | SOURCE | IDENTIFIER |
| --- | --- | --- |
| Antibodies | | |
| Rabbit anti-Ptbp1 | Abcam | Cat#ab133734; RRID: AB_2814646 |
| Rabbit anti-Iba1 | Abcam | Cat#ab289874; RRID: AB_2942069 |
| Mouse anti-GFAP | Abcam | Cat#ab279290; RRID: AB_2920668 |
| Rabbit anti-Islet1 | Abcam | Cat#ab109517, RRID: AB_10866454 |
| Mouse anti-DCX | Cell Signaling | Cat#14802; RRID: AB_2798619 |
| Rabbit anti-Map2 | Cell Signaling | Cat#8707; RRID: AB_2722660 |
| Rabbit anti-β3-tubulin | Cell Signaling | Cat#5568; RRID: AB_10694505 |
| Mouse anti-NeuN | Cell Signaling | Cat#94403; RRID: AB_2904530 |
| Rabbit anti-S100a10 | ABclonal | Cat#A1987; RRID: AB_2764013 |
| Rabbit anti-C3 | Abcam | Cat#ab97462; RRID: AB_10679468 |
| Rabbit anti-ntng2 | Novus Biologicals | Cat#NBP2-68828; RRID: AB_3354090 |
| Rabbit anti-lrrc4 | ThermoFisher |  |
| FITC-AffiniPure Goat Anti-Mouse IgG (H+L) | Jackson ImmunoResearch Labs | Cat#115-095-003; RRID: AB_2338589 |
| FITC-AffiniPure Goat Anti-Rabbit IgG (H+L) | Jackson ImmunoResearch Labs | Cat#111-095-003; RRID: AB_2337972 |
| Cy3-AffiniPure Goat Anti-Mouse IgG (H+L) | Jackson ImmunoResearch Labs | Cat#115-165-003; RRID: AB_2338680 |
| Cy3-AffiniPure Goat Anti-Rabbit IgG (H+L) | Jackson ImmunoResearch Labs | Cat#111-165-003; RRID: AB_2338000 |
| Goat anti-Mouse IgG1 Cross-Adsorbed Secondary Antibody, Alexa Fluor™ 647 | ThermoFisher | Cat#A-21240, RRID: AB_2535809 |
| Bacterial and virus strains | | |
| AAV-GFAP-CasRx-control | Zhou et al. [28](file:///E:/投稿/nc/manuscript.docx#_ENREF_28) | RRID: Addgene_154000 |
| AAV-GFAP-CasRx-Ptbp1 | Zhou et al. [28](file:///E:/投稿/nc/manuscript.docx#_ENREF_28) | RRID: Addgene_154001 |
| Biological samples | | |
| Mouse spinal cord tissue | This study | N/A |
| Mouse DRG tissue | This study | N/A |
| Mouse sciatic nerve tissue | This study | N/A |
| Chemicals, peptides, and recombinant proteins | | |
| Neurobasal Medium | Life Technologies | Cat#21103049 |
| N2 supplement | Gibco | Cat#17502048 |
| B27 supplement | Gibco | Cat#5001207 |
| DMEM/F12 | Gibco | Cat#11039021 |
| Deposited data | | |
| RNA-sequencing data | PRJNA1069664 | <https://www.ncbi.nlm.nih.gov/sra/PRJNA1069664> |
| ATAC-sequencing data |  |  |
| Experimental models: Cell lines | | |
| Primary astrocytes | This study | N/A |
| Satellite glial cells | This study | N/A |
| Experimental models: Organisms/strains | | |
| Mouse: Adult C57BL6/J | Shanghai Jihui Laboratory Animal Breeding Company | N/A |
| Oligonucleotides | | |
| siRNA sequence: Supplementary Table 1 | This study | N/A |
| PCR primers: *Ptbp1* forward: GCCAGAACATCTACAACGCC | This study | N/A |
| PCR primers: *Ptbp1* reverse: CCGGGCGCACCAAAGG | This study | N/A |
| PCR primers: *GDNF* forward: GATTCGGGCCACTTGGAGTT | This study | N/A |
| PCR primers: *GDNF* reverse: TCTTCAGGCATATTGGCGGC | This study | N/A |
| PCR primers: *GAPDH* forward: CTTCATTGACCTCAACTACATGG | This study | N/A |
| PCR primers: *GAPDH* reverse: GAGATGATGACCCTTTTGGC | This study | N/A |
| PCR primers: *S100a10* forward: CTTGACAAAGGAGGACCTGAGAGTG | This study | N/A |
| PCR primers: *S100a10* reverse: CCCACTTTTCCATCTCGGCACTG | This study | N/A |
| PCR primers: *GFAP* forward: GCGAAGAAAACCGCATCACC | This study | N/A |
| PCR primers: *GFAP* reverse: AAGGGAGAGCTGGCAGG | This study | N/A |
| PCR primers: *Clcf1* forward: AGCAGGAAGAACCTGCCAAA | This study | N/A |
| PCR primers: *Clcf1* reverse: CCCACGAGTCCCCTCTAGTC | This study | N/A |
| PCR primers: *Serping1* forward: CCCACGAGTCCCCTCTAGTC | This study | N/A |
| PCR primers: *Serping1* reverse: TTGGACCAGGTTTGGACGCA | This study | N/A |
| PCR primers: *Fbln5* forward: CTGGGAATGCACAGCAGCAG | This study | N/A |
| PCR primers: *Fbln5* reverse: TACCCGCCATTCTGGTTGAC | This study | N/A |
| Software and algorithms | | |
| Image J | <https://imagej.nih.gov/ij/> | RRID: SCR_002285 |
| Prism 8 | <https://www.graphpad.com/> | N/A |
| Abobe Illustrator | <https://www.adobe.com/products/> | N/A |
